# Delayed Nerve Stimulation Promotes Axon-Protective Neurofilament Phosphorylation, Accelerates Immune Cell Clearance and Enhances Remyelination *In Vivo* in Focally Demyelinated Nerves

**DOI:** 10.1371/journal.pone.0110174

**Published:** 2014-10-13

**Authors:** Nikki A. McLean, Bogdan F. Popescu, Tessa Gordon, Douglas W. Zochodne, Valerie M. K. Verge

**Affiliations:** 1 CMSNRC (Cameco MS Neuroscience Research Center) and Department of Anatomy and Cell Biology, University of Saskatchewan, Saskatoon, SK, Canada; 2 Department of Surgery, Division of Plastic Reconstructive Surgery, University of Toronto, Toronto, ON, Canada; 3 Department of Medicine, Division of Neurology, University of Alberta, Edmonton, AB, Canada; Aix Marseille University, France

## Abstract

Rapid and efficient axon remyelination aids in restoring strong electrochemical communication with end organs and in preventing axonal degeneration often observed in demyelinating neuropathies. The signals from axons that can trigger more effective remyelination *in vivo* are still being elucidated. Here we report the remarkable effect of delayed brief electrical nerve stimulation (ES; 1 hour @ 20 Hz 5 days post-demyelination) on ensuing reparative events in a focally demyelinated adult rat peripheral nerve. ES impacted many parameters underlying successful remyelination. It effected increased neurofilament expression and phosphorylation, both implicated in axon protection. ES increased expression of myelin basic protein (MBP) and promoted node of Ranvier re-organization, both of which coincided with the early reappearance of remyelinated axons, effects not observed at the same time points in non-stimulated demyelinated nerves. The improved ES-associated remyelination was accompanied by enhanced clearance of ED-1 positive macrophages and attenuation of glial fibrillary acidic protein expression in accompanying Schwann cells, suggesting a more rapid clearance of myelin debris and return of Schwann cells to a nonreactive myelinating state. These benefits of ES correlated with increased levels of brain derived neurotrophic factor (BDNF) in the acute demyelination zone, a key molecule in the initiation of the myelination program. In conclusion, the tremendous impact of delayed brief nerve stimulation on enhancement of the innate capacity of a focally demyelinated nerve to successfully remyelinate identifies manipulation of this axis as a novel therapeutic target for demyelinating pathologies.

## Introduction

Myelination is crucial for proper neurological function, with damage to the myelin sheath having potentially devastating consequences. While the nervous system has a moderate innate capacity for remyelination, this process is far from perfect. Demyelination and inflammation are common features of a variety of peripheral and central nervous system disorders, including Guillain-Barré syndrome and multiple sclerosis respectively [1], [2], [3]. Despite an increased capacity for peripheral nerve remyelination in patients with Guillain-Barré syndrome [2], [4], many are plagued by residual impairment [4], [5] as effective remyelination and repair of focally demyelinated nervous tissue is fraught with challenges.

One of these challenges is the increased loss of axons observed in demyelinating disorders, believed to be due to the vulnerability of the demyelinated axon to degenerative processes (reviewed in [6], [7]). This loss may be linked to alterations in neurofilament proteins that serve important roles in the radial growth of axons [8], [9] thereby impacting axonal caliber and conduction efficiency [10]. Further, myelinated axons normally display high levels of medium and high molecular weight neurofilament phosphorylation [11]. Neurofilament phosphorylation increases the axonal caliber [12] important in determining the onset of myelination [13]. It is also protective to the axon, with dephosphorylated neurofilaments susceptible to proteolysis by the calcium-dependent protease calpain [14], [15], [16], while phosphorylated neurofilaments are resistant to degradation [17]. Importantly, neurofilament phosphorylation is controlled by the myelination process [18] and upon demyelination, these filaments become dephosphorylated, both in experimental models of dysmyelination [19] and in human demyelinating disease states [20].

Disruption in the organization of the nodes of Ranvier is another critical feature of segmental and paranodal demyelination. Typically, nerves display a distinct and restricted regional distribution of the protein Caspr and the Kv1.2 potassium channel in the paranodal and juxtaparanodal regions respectively, which is lost following demyelination and can assume a more diffuse axonal distribution [21], [22]. Recapitulation of the distinct nodal architecture is key during the remyelination process [23] and together with neurofilament phosphorylation serve as important measures of the efficiency of repair processes and axonal health, respectively. Thus, remyelination strategies that also target these events would be beneficial.

A number of additional cellular events must occur for effective remyelination, including the clearance of the myelin debris [24], [25], [26]. Monocyte lineage cells, such as macrophages and microglial cells, are largely responsible for this clearance [27], [28], [29], [30], with enhanced myelin debris clearance being linked to improved remyelination [25]. Axonal injury or demyelination also drives Schwann cell dedifferentiation and the acquisition of a reactive phenotype, characterized by prominent glial fibrillary acidic protein (GFAP) expression [31]. The rapid differentiation of these Schwann cells back to a myelinating phenotype is desirable. This can be impacted by increased expression of molecules such as neurotrophins, best known for their roles in neuronal growth, survival and regeneration [32], [33]. Specifically, in the peripheral nervous system, the neurotrophin brain-derived neurotrophic factor (BDNF) is important for initiation of the myelination program [34], [35] primarily via activation of the p75 neurotrophin receptor expressed by Schwann cells [36]. Sources for this activation include neurons [37], [38], [39], [40], [41], Schwann cells [42], [43], or the infiltrating activated macrophages [44], [45]. This results in an accumulation of BDNF at the site of demyelination that appears to be crucial for enhancing the remyelination process.

Remyelination can occur in response to damage to the myelin sheath of peripheral [46], [47] or central nervous system axons [48], although it is less effective in the latter. However, the issue of how to promote early and rapid activation of this response following a demyelinating insult remains unresolved. Increased neuronal electrical activity may serve as an important intrinsic signal for remyelination [49]. For example, in a different pathology, namely peripheral nerve injury, the immediate brief electrical stimulation of the nerve proximal to the injury at the time of nerve injury and repair (ES; 1 hour (hr) at 20 Hz) accelerates the growth of both motor and sensory axons across the repair site [50], [51], [52], [53] and is associated with enhanced remyelination of the regenerating axons [54]. The improved regeneration is associated with increases in the neuronal expression of both BDNF and its receptor trkB [55], and neurotrophin signaling [56], [57]. These findings raise the question of whether the beneficial effects of brief ES will still be apparent in an experimental model of demyelinating disease in which axons have been focally demyelinated but remain intact, such as the highly-studied model of lysophosphatidyl choline (LPC)-induced demyelination [58], [59]. This model largely spares axonal structure [60], as compared to crush or transection injury models.

In demyelinating disease, patients will present neurological deficits only after the demyelination has occurred, leaving one unable to therapeutically intervene at the initiation of the demyelinating event. Thus, we hypothesized that delayed brief nerve ES (1 hour at 20 Hz) delivered proximal to a site of focal demyelination induced five days prior, will induce cellular changes that result in more effective remyelination. Here, we present evidence that delayed brief ES does indeed have a facilitatory effect on remyelination, helping the nerve overcome many of the barriers and molecular challenges associated with this event.

## Materials and Methods

### Surgical Procedures

All procedures were approved by the University of Saskatchewan Animal Research Ethics Board and adhered to the Canadian Council on Animal Care guidelines for humane animal use. Animals were given buprenorphine (Temgesic; 0.05– 0.1 mg/kg) subcutaneously pre- and postoperatively to minimize incisional discomfort. A total of 80 young adult male Wistar rats (150–200g) were used in the study. Rats were deeply anaesthetized with inhaled isoflurane (2% delivered at a rate of 2L/min). To create a unilateral focal demyelinating lesion, the right sciatic nerve was exposed at the point of trifurcation into the common peroneal, tibial and sural branches and 2 µl of a 1% LPC/1% Fluorogold (FG; Fluorochrome Inc. Denver, CO, USA) solution was injected into the tibial branch of the sciatic nerve, just distal to the trifurcation, using a 20–30 micron tip glass needle connected to a Hamilton syringe. FG served to demarcate the demyelination zone, while an epineural 10-0 suture marked the injection site.

Five days later, the animals were randomly assigned to experimental groups. In all groups except the 5-day post LPC group (which serves as the demyelination baseline group), half of the animals were anaesthetized and the right sciatic nerves re-exposed to perform ES. Insulated stainless steel wires bared of insulation (2–3 mm for the anode, 5–10 mm for the cathode) were connected to a Grass (Quincy, MA) SD-9 stimulator. The cathodic wire was wrapped around the exposed nerve, 2–3 mm proximal to the injection site. The anode was placed between the skin and muscle. ES was performed as previously described by Al-Majed et al. [50]. A Grass stimulator delivered a continuous 20 Hz train of supramaximal pulses (100 msec; 3V) for one hour. Epineural 10-0 suture marked the stimulation site. These stimulation parameters employed in this study were selected as they closely mimic the firing patterns of both motor and sensory neurons [50], [61].

In addition to the LPC-injected +/- ES animals, the following controls were generated: naïve rats (n = 3); naïve animals with brief ES as above (n = 3); LPC-injected animals with sham stimulation where electrodes were connected but the stimulator was not turned on (n = 3); and LPC-injected animals treated with 2% lidocaine soaked gelfoam applied to the sciatic nerve proximal to ES 30 minutes prior to and during ES (n = 3) followed by thorough rinsing with sterile PBS before closing the incision.

Animals were processed for analysis at various time points post-injection with stimulated groups being paired with non-stimulated groups in the same cryomolds to ensure processing under identical conditions (5 days, n = 6; 6 days, n =  6 LPC and 6 LPC+ES; 8 days, n = 8 LPC and 8 LPC+ES; 10 days, n = 8 LPC and 8 LPC+ES; and 12 days, n = 11 LPC and 12 LPC+ES). For immunohistochemistry, animals were euthanized with Euthanyl Forte overdose (Bimeda-MTC, Cambridge, ON) and transcardially perfused with 4% paraformaldehyde. One cm of ipsilateral sciatic nerve (bordering both sides of the sites of LPC injection and electrical stimulation) and equivalent contralateral were removed, post-fixed and cryoprotected overnight in 20% sucrose. Tissues were embedded in cryomolds with OCT compound (Tissue Tek, Miles INC, Elkhart, IN) frozen in cooled isopentane and stored at −80°C until processing. For fresh-frozen immunohistochemistry, animals were euthanized with Euthanyl Forte (75 mg/kg) and transcardially perfused with 60 mL of ice-cold phosphate buffered saline (PBS). Tissue was removed, immediately embedded and frozen (as above) and stored at −80°C until processing.

### ELISA

To generate tissue samples for the ELISA, a separate cohort of animals received LPC focal demyelinating lesions with or without brief ES as above (n = 16 animals total with 2 animals per condition with 2 naïve rats and 5d LPC contralateral nerves serving as controls). Animals were euthanized at 5, 8, 10 and 12 days post-LPC. One cm lengths of sciatic nerve bordering both sides of the demyelinating lesion equally were removed along with corresponding levels of control nerves, placed in lysis buffer (137mM NaCl, 20mM Tris-HCl (pH 8.0), 1% NP40, 10% glycerol, 1mM PMSF, 10 µg/mL aprotinin, 1 µg/mL leupeptin, 0.5mM sodium vanadate) and stored at −80°C until processing. BDNF content was measured using the BDNF E_max_ Immunoassay System (Promega, Madison, WI) according to the manufacturer's protocol. Plates were immediately read at a wavelength of 450 nm in a SpectraMax 340 (Molecular Devices, Sunnyvale, CA) spectrophotometric plate reader equipped with SoftMax Pro 5 software (Molecular Devices, Sunnyvale, CA).

### Western Blotting

Surgical procedures were performed as above on a separate cohort of animals (n = 18 animals total with 3 LPC and 3 LPC+ES animals per time point). Animals were euthanized at 5, 8 and 10 days post-LPC injection. Three naïve animals served as controls. A one cm segment of sciatic nerve bordering both sides of the injection site equally was removed from each rat, along with corresponding levels of contralateral and control naïve nerves. The nerve segments were placed in lysis buffer (137mM NaCl, 20mM Tris-HCl (pH 8.0), 1% NP40, 10% glycerol, 1mM PMSF, 10 µg/mL aprotinin, 1 µg/mL leupeptin, 0.5mM sodium vanadate), homogenized and stored at −80°C until processing. Extracted proteins from pooled samples (N = 3 rats/experimental condition/sample) at each time point and protein molecular size markers (Licor, catalog #928-40000) were separated by SDS-PAGE (10% acrylamide gels) and transferred to PVDF membranes (Bio-Rad, catalog # 162-0177) by electroblotting. Membranes were blocked overnight in Licor Odyssey blocking buffer (catalog # 927-40000) at 4°C with gentle agitation. Primary antibodies mouse anti-CD68 (ED-1; 1∶500, Cedarlane, catalog # MCA341R); mouse anti-phosphorylated neurofilament (SMI-31, 1∶1000, Covance, catalog # SMI-31R); mouse anti-pan-neurofilament antibody (NF; 1∶1000, Dako, catalog # M0762); and mouse anti-β III tubulin (loading control; 1∶100, Millipore, catalog # MAB1637) in PBS with 0.05% Tween 20 were applied at 4°C for 72 hours with gentle agitation. Membranes were washed in PBS with 0.05% Tween 20 and infrared dye-conjugated secondary antibody (donkey anti-mouse IR-800 (1∶5000, Licor, catalog # 926-32212)) was applied for one hour at room temperature. Membranes were washed in PBS with 0.05% Tween 20, followed by PBS before scanning on a Licor Odyssey 9120 infrared scanning system.

BDNF Western blots were conducted under nonreducing conditions (β-mercaptoethanol omitted) using a rabbit anti-BDNF antibody that recognizes both pro- and mature-BDNF (Biosensis, catalog # R-083-100) with donkey anti-rabbit IR-800 (1∶5000, Licor, catalog # 926-32213) used to visualize the bands.

### Histochemistry

#### Immunofluorescence

Longitudinal sections of fixed frozen nerve tissue cut at 10 µm on a Microm cryostat were thaw-mounted onto silanized glass slides. Slides were air-dried for 15 minutes and washed in PBS prior to blocking in 10% normal donkey serum and 0.1% Triton X-100 in PBS at room temperature. Primary antibodies diluted in 2% normal donkey serum and 0.1% Triton X-100 in PBS were applied overnight at 4°C in a humidified chamber. Primary antibodies included: mouse anti-myelin basic protein (MBP; 1∶250, Covance, catalog # SMI-94R); mouse anti-CD68 (ED-1; 1∶250, Cedarlane, catalog # MCA341R); rabbit anti-glial fibrillary acidic protein (GFAP; 1∶800, Dako, catalog # Z 0334); mouse anti-beta III tubulin (β-III; 1∶100, Millipore, catalog # MAB1637); rabbit anti-β-III (1∶1000, Sigma-Aldrich, catalog # T 2200); chicken anti-BDNF (1∶1000, Promega, catalog # G1641); mouse anti-phosphorylated neurofilament (SMI-31, 1∶1000, Covance, catalog # SMI-31R). Slides were washed and secondary antibodies diluted in PBS applied for one hour at room temperature - DyLight 488-conjugated donkey anti-mouse IgG (1∶200, Jackson ImmunoResearch); Cy3-conjugated donkey anti-rabbit IgG (1∶300, Jackson ImmunoResearch). Slides were washed in PBS and mounted with a coverslip using 50% glycerol/PBS.

For fresh frozen immunohistochemistry, tissue was cut as above and slides were immediately fixed in 4% paraformaldehyde for 15 minutes at room temperature, washed in PBS, and blocked for 1hr at room temperature in 10% normal donkey serum and 0.3% Triton X-100 in PBS. Primary antibodies diluted in 2% normal donkey serum and 0.3% Triton X-100 in PBS were incubated overnight at 4°C in a humidified chamber. Primary antibodies included: mouse anti-potassium channel Kv 1.2 (Kv1.2; 1∶1000, Millipore, catalog # MABN77); rabbit anti-Contactin associated protein (Caspr; 1∶4500, Abcam, catalog # ab34151). Secondary antibodies diluted in PBS were applied for one hour at room temperature. Secondary antibodies included DyLight 488-conjugated donkey anti-mouse IgG (1∶200, Jackson ImmunoResearch); Cy3-conjugated donkey anti-rabbit IgG (1∶300, Jackson ImmunoResearch). Slides were washed in PBS and mounted with a coverslips using 50% glycerol/PBS.

#### DAB Immunohistochemistry

Longitudinal sections of fixed frozen tissue mounted on silanized slides were dried overnight at 37°C, washed in PBS and endogenous peroxidase activity quenched with 0.3% hydrogen peroxide for 30 minutes at room temperature. Slides were blocked in PBS containing 10% fetal bovine serum (FBS). Mouse monoclonal pan-neurofilament antibody (NF) recognizing the 70 kDa subunit common to all 3 neurofilament chains was diluted 1∶800 in 3% horse serum & 10% FBS and incubated overnight at 4°C, Dako # M0762). Slides were washed in PBS and biotinylated mouse secondary antibody (Amersham, catalog # RPN1001) diluted 1∶200 in 10% fetal bovine serum in PBS was applied for one hour at room temperature. Slides were washed and HRP-conjugated avidin applied for one hour at room temperature before developing with DAB.

#### Histological stains

Sections processed for DAB immunohistochemistry also underwent staining with the Luxol Fast Blue method combined with Nuclear Fast Red counterstain to detect the presence of myelin. Sections were dehydrated through an alcohol gradient and cleared in xylene prior to mounting with a coverslip using Permount (Fisher Scientific, catalog # SP15-500). Slides were analysed on an Olympus BX53 microscope and images digitally captured using cellSens Standard software (Olympus).

### Data Analysis

#### Histochemical

To ensure accurate analysis of relative changes in IF signal between experimental groups, nerve segments ipsilateral and contralateral to LPC injection from both experimental and control groups were always mounted on the same slide so that processing was conducted under identical conditions. Immunofluorescence data was gathered from digital images of the site of demyelination captured under identical exposure conditions using Northern Eclipse v7.0 software (EMPIX Imaging Inc.) and a Zeiss Axio Imager M.1 fluorescence microscope. Demyelinated regions of interest were identified by the presence of FG-positive staining. Analysis was carried out by tracing the outline of the FG-positive region of interest using Northern Eclipse, which then calculates the Average Gray and total area (in microns^2^) for the image, yielding average Gray per micron^2^. For each time point examined, all values obtained at that time point were normalized to the mean value of the Average Gray per micron^2^ value for the nerves ipsilateral to LPC treatment for the *LPC Only* animals at that time point. The relative fluorescence signal for each marker with and without ES was compared using the Kruskal-Wallis one-way ANOVA with Dunn's post-test analysis (6, 8, 10 or 12 days post-injection) or Student's t-test (5 days post-injection). Results achieved statistical significance at p <0.05.

#### ELISA

BDNF protein levels in samples of sciatic nerve assessed via ELISA were determined by interpolation from the standard curve included in the assay, followed by background correction (subtracting the average background OD_450_ from that of each of the experimental and standard curve). For each sample the mean BDNF concentration was determined for replicate wells. Statistical analysis was performed using a one-way ANOVA with Tukey's multiple comparison test. Results achieved statistical significance at a p value <0.05.

#### Western blot

Data (from two replicates using protein isolated from pooled nerves from 3 animals per experimental group) was analyzed using the ImageJ software application. Mean densitometry values for all experimental conditions were normalized to the β-III tubulin loading control and expressed as a fold difference of the mean densitometry reading for two lanes of protein extract from naïve animals run on the same gel. Student's t-tests were performed to determine the significance of changes relative to naïve controls for each protein of interest (CD68/ED-1; NFM; or phosphorylated NFM) at each time point with and without ES. Results achieved statistical significance at p <0.05.

## Results

### Identification of focal demyelination zone

**Figure 1:**
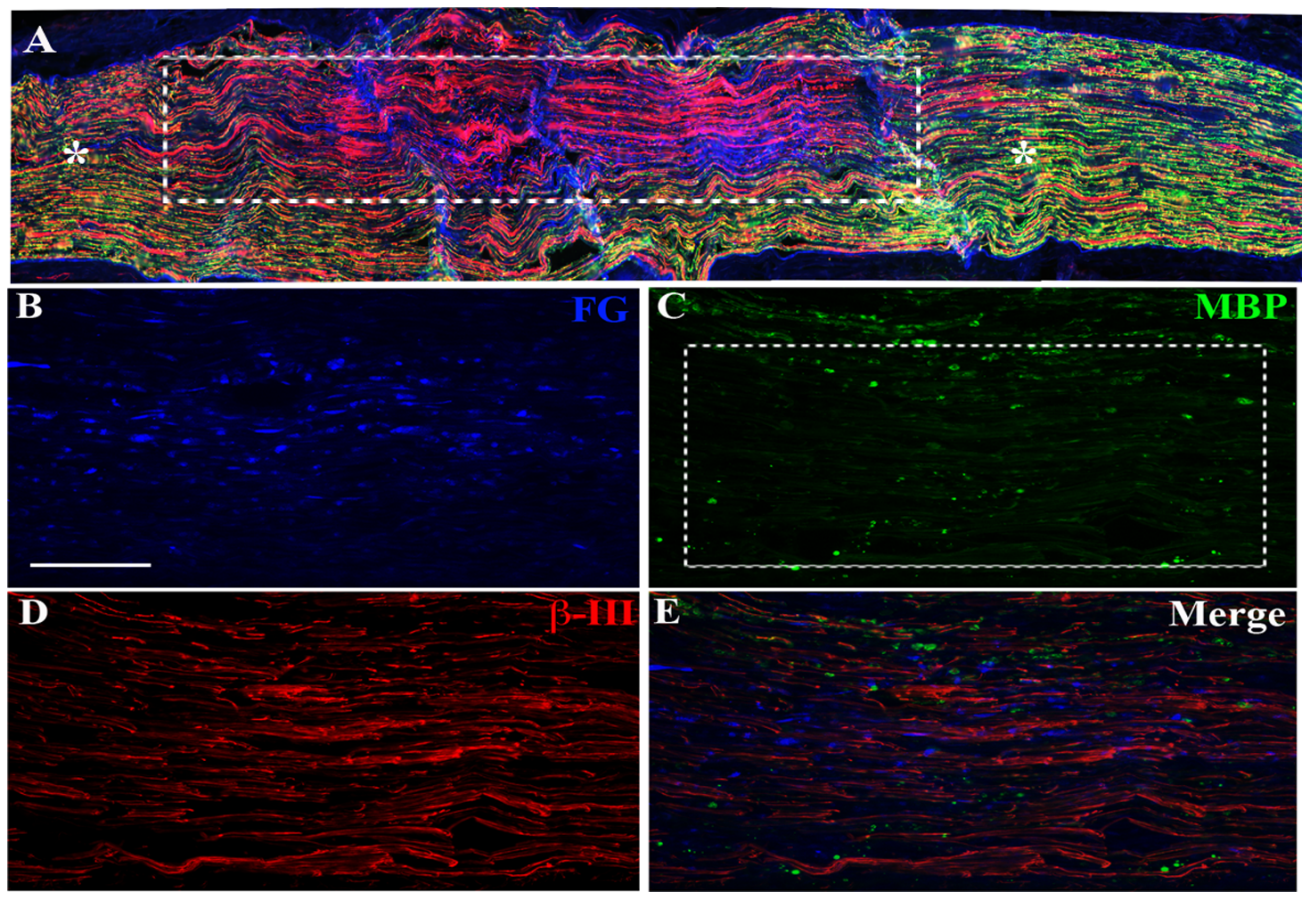
FluoroGold (FG) staining delineates the demyelination zone. Representative fluorescence photomicrographs from a longitudinal section of tibial nerve six days after injection with a lysophosphatidyl choline (LPC)/FG mixture and doubly immunostained for myelin basic protein (MBP; green, C) and β-III tubulin (red, D). Note: FG was taken up by cells in the injection site region and diffused beyond the injection site. There was good register between the most intensely stained region of FG (blue, A, E) and the area of demyelination as defined by the punctate immunostaining for MBP and a lack of the normally uniform linear MBP staining (A, C). The presence of positive linear β-III tubulin IF indicates that axons within the demyelination site persist (A, D). The demyelination site was identified by the joint presence of FG, axonal (β-III tubulin) and paucity of myelin marker (MBP) (boxed areas A, E) in contrast to the robust MBP immunostaining outside the zone of demyelination (asterisks, A). Thus, FG serves to identify the regions of interest (ROIs; similar to that outlined by dashed lines in (C)), where alterations in various markers impacted by the LPC and electrical stimulation were quantified (see Figures 2, 4, 5, 7). Scale bar = 100 µm.

The ability to clearly demarcate the initial region of demyelination in the affected nerve is necessary to reliably assess alterations in cellular events and proteins within zones of demyelination and remyelination. Thus, the retrograde fluorescent tracer Fluorogold (FG) was co-injected with the demyelinating agent lysophosphatidyl choline (LPC) into the tibial nerve creating a readily identifiable injection zone and subsequent region of demyelination and remyelination. While some FG was retrogradely transported back to the neuronal cell bodies contributing axons to the tibial branch, sufficient FG remained at the zone of demyelination to readily demarcate it. The 5 day post-LPC demyelination site was readily identified by the joint presence of FG (Figure 1B), diminished myelin basic protein (MBP; a component of myelin Figure 1C) immunofluorescence (IF) and IF for β-III tubulin, an axonal marker (Figure 1D). There was tight register between the region of demyelination 5 days post-LPC and the co-injected FG (Figure 1B and Figure 1C), as shown by a relative lack of MBP in the most FG intense region. These FG intense regions were used to create regions of interest (ROIs) in which the intensity of immunofluorescence signal for individual markers examined in this study were quantified. For all sections examined, the loss of MBP signal did not extend beyond the FG-positive ROI, with consistent demyelination observed in the FG-defined ROI at 5 days (d) post-LPC (Figure 1A). There was also no evidence of a contralateral effect in response to ES, as the contralateral tibial nerves did not differ from naïve controls with respect to the various markers examined (data not shown). ES did not appear to impact the integrity of the nerve, as levels of β-III tubulin immunofluorescence signal over sections of naïve nerve were not discernibly different from sections of nerve that had undergone only brief ES (no demyelination) seven days previous. Finally, the impact of ES on the parameters examined in this study were likely due to neuronal activation, because they were abolished when lidocaine was applied to the tibial nerve proximal to the stimulation site just prior to and during the one hour ES period, while sham ES (electrodes put in place on the nerve, but not turned on) had no effect (Figure S1; Figure S2; Figure S3).

### Brief ES increases myelin basic protein (MBP) expression

**Figure 2:**
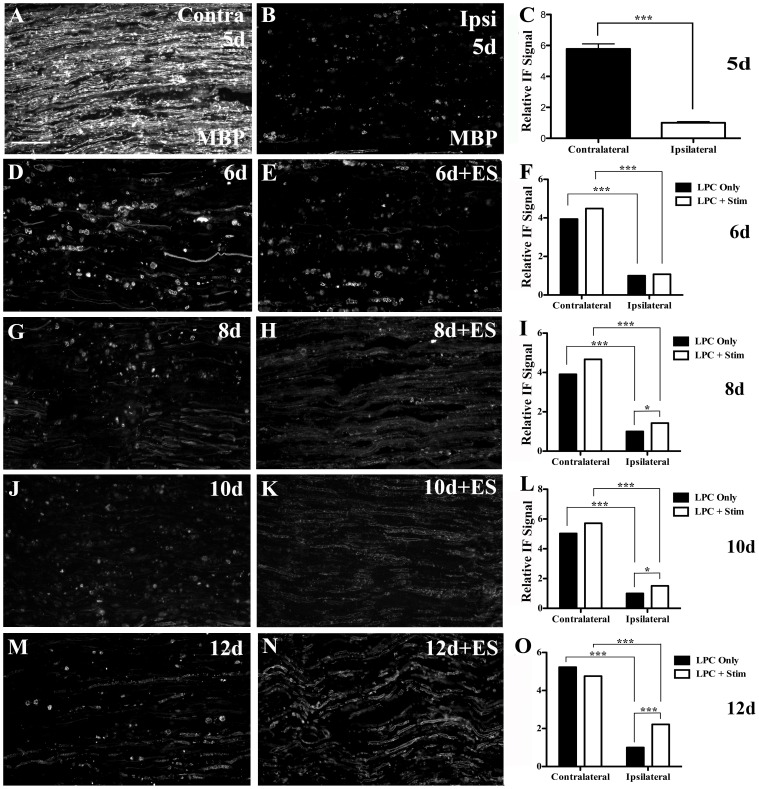
Increased myelin basic protein (MBP) expression following 1hr ES delivered 5 d post-LPC. Representative photomicrographs from FG-positive areas on longitudinal sciatic nerve sections processed for MBP immunofluorescence (IF) reveal extensive demyelination 5d post lysophosphatidyl choline (LPC)/FG demyelinating injection (B) as compared to *contralateral* normal control nerves (A). Temporal analysis of LPC) focal demyelination +/- electrical stimulation (ES) as indicated in days post-LPC: 6d (D), 6d+ES (E), 8d (G), 8d+ES (H), 10d (J) 10d+ES (K) and 12d (M) 12d+ES (N) post-LPC. Note: In the LPC only group, MBP IF was localized in distinct patches consistent with uptake by phagocytosing cells (D, G, J) with faint linear regions similar to periaxonal MBP localization observed by 12 days post-LPC only injection (M). In contrast, in the LPC + ES group faint regions of MBP peri-axonal-like IF were already apparent 8d post-LPC (3d post-ES; H) and consistently stronger in the 10d post-LPC (5d post-ES; K) and 12d post-LPC (7d post-ES; N). Summary bar graphs of relative changes in immunofluorescence signal for MBP in regions of demyelination (C, F, I, L, O). Note: values obtained from individual slides at each time point were normalized to the mean value of the Average Gray per micron^2^ readings for the nerves ipsilateral to LPC treatment for the LPC only animal on that slide and for that time point. N = 4-6 animals analyzed per condition; regions quantified per condition: 49 ipsi and 38 contra (5d, LPC only); 128 ipsi and 76 contra (6d, LPC only); 81 ipsi and 54 contra (6d LPC+Stim); 83 ipsi and 49 contra (8d, LPC only); 60 ipsi and 58 contra (8d, LPC+Stim); 62 ipsi and 52 contra (10d, LPC only); 54 ipsi and 48 contra (10d, LPC+Stim); 155 ipsi and 73 contra (12d, LPC only); 119 ipsi and 104 contra (12d LPC+Stim). Asterisks indicate significant differences between experimental groups; *P<0.05, **P<0.01, ***P<0.001. Scale bar  =  100 µm.

Injection of LPC into the tibial branch of the sciatic nerve induced a rapid, focal demyelinating lesion. By five days post-LPC injection, a near complete demyelination of the axons within the injection site was observed (Figure 1, 2B). There was clear disruption in the normal linear pattern of MBP IF (Figure 2A), consistent with segmental demyelination. The MBP IF signal within the demyelination zone was greatly diminished and when detected was punctate, likely representing myelin debris that was either free within the endoneurium or that had been taken up by phagocytic macrophages. Analysis comparing the relative alterations in MBP IF signal over contralateral versus ipsilateral tibial nerves revealed a significant decrease in MBP, consistent with myelin loss in the LPC-injected (ipsilateral) nerves (Figure 2C).

Alterations in MBP expression induced by the 1 hr ES treatment (applied at 5d post-LPC injection) were assessed relative to nerves that had undergone LPC-induced demyelination only at the same time point following ES. At 6d post-LPC (1d post-ES), the levels of MBP detected in the focally demyelinated tibial nerves of animals were not significantly different between the experimental group that received ES and those that did not (Figure 2D & 2E). The lesion sites in both groups were nearly devoid of linear MBP signal and differed significantly from the intact contralateral nerve (Figure 2F).

By 8d post-LPC injection (3d post-ES), however, the stimulated nerves were significantly different and displayed more intense MBP IF signal. This included less visible MBP-positive myelin debris and a more linear pattern of MBP signal in the ES-treated nerves, suggestive of early segmental remyelination (Figure 2G, H, I). While the stimulated and non-stimulated groups did not differ greatly in the level of MBP IF detected 10d post-LPC (Figure 2J, K, L; 5d post-ES), there was a distinct difference in the localization of the IF signal. It appeared linear in the ES group as opposed to its localization to predominantly small round cells consistent with monocytes, in the LPC only nerves. By 12d post-LPC the ES group was vastly different from the control LPC only group (Figure 2M, N, O; 7d post-ES). At this last time point examined, nerves receiving ES had clear evidence of greater remyelination than controls, albeit incomplete.

### Accelerated Node of Ranvier reorganization in electrically stimulated demyelinated nerves

**Figure 3:**
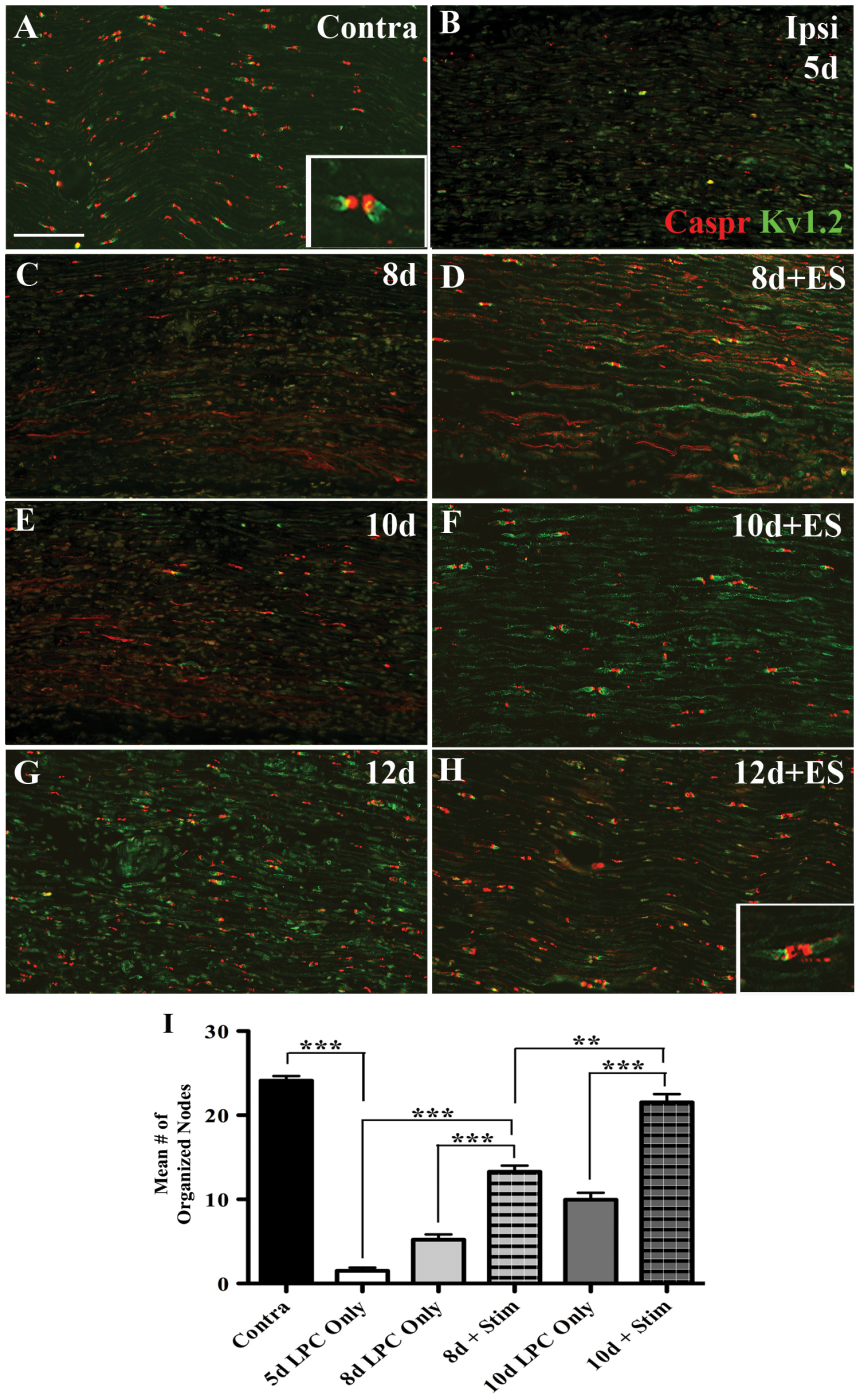
Accelerated node of Ranvier reorganization in focally demyelinated nerves subjected to 1hr ES. Representative photomicrographs of FG-positive (i.e focally demyelinated) regions of longitudinal tibial nerve sections dually immunostained for the paranodal protein Caspr (red) and the juxtaparanodal Kv1.2 ion channel (green). Contralateral control nerves display well-organized nodes of Ranvier with Caspr IF in the paranodal region and Kv1.2 IF at the juxtaparanodal region (A, insert reveals nodal staining at higher magnification) and an average of 24.1 visible nodes per field of view as defined by a 1300×900 pixel rectangle superimposed on the photomicrograph (I). A marked loss of nodal organization was observed 5d post-lysophosphatidyl choline (LPC)/FG injection, with an average of 1.5 nodal regions per field of view (B, I). Temporal analysis of LPC +/- ES delivered 5 d post-LPC and indicated in days post-LPC: 8d (C), 8d+ES (D), 10d (E), 10d+ES (F) and 12d (G) 12d+ES (H) post-LPC, revealed that ES delivered 5 d post_LPC resulted in nodal reorganization apparent as early as 8d post-LPC (8d+ES - 3d post-ES; D), with a mean of 13.28 nodes per field of view, as compared to 5.19 in the non-stimulated nerves (8d post-LPC only; C, I). The reorganization continued at 10d post-LPC in the ES nerves, approaching contralateral control nerve levels (10d+ES - 5d post-ES; F), with a mean of 21.53 nodes per field of view in the stimulated nerves (I), compared to 9.96 in the non-stimulated (10d post-LPC; E, I) that did not discernibly change at the 12d post-LPC (12d+ES - 7d post-ES; H and insert). Tissue from the LPC only group displayed modest nodal re-organization (G)12d post-LPC consistent with the appearance of only faint remyelination (see Figure 2M). Scale bar  =  100 µm.

The node of Ranvier has a highly structured molecular organization, with the Nav1.6 sodium channels clustered at the node [62], flanked by the contactin-associated protein (Caspr) at the paranode, and voltage gated potassium channels (including Kv1.2) in the juxtaparanodal region [21]. Axonal demyelination resulted in a loss of the discrete regional localization of the two node of Ranvier associated markers. Both paranodal Caspr and juxtaparanodal Kv1.2 assumed a diffuse distribution with the two markers often colocalized (Figure 3B, C, E), instead of being highly localized and distinct from each other ([21]; Figure 3A and insert, Figure 3A). At 5d post-LPC injection there was nearly a complete loss of the distinct restricted Caspr and Kv1.2 staining, with an average of 1.5 visible organized nodal regions in the fields of view examined, compared to 24.1 nodal regions in an equivalent field in the contralateral uninjured nerve. Disruption of the nodal organization largely persisted in both the ES and non-stimulated nerves at 8d post-LPC injection (3d post-ES; Figure 3C, D). At this time point however, early evidence of reorganization of nodal regions began to emerge, especially in the ES group (Figure 3D), with an average of 13.28 Caspr-positive nodal regions observed per field of view, compared to 5.19 for the non-stimulated nerves (Figure 3I). By 10d post-LPC (5d post-ES) the incidence of Caspr- and Kv1.2-positive organized nodal regions in the stimulated nerves (Figure 3F) was equivalent to that observed in the normal nerve with an average of 21.53 distinct Caspr-positive nodal regions per field of view (Figure 3I). However, despite this, there was still some faint diffuse Kv1.2 staining in the axons. This differed markedly from the non-stimulated nerves that continued to display a disorganized, predominantly diffuse pattern of Caspr- and Kv1.2-positive IF signal (Figure 3E) with an average of only 9.96 visible Caspr-positive nodal regions per field of view (Figure 3I). By 12d post-LPC (7d post-ES) there is a return to the normal pattern of Caspr and Kv1.2 localization in ES nerves (Figure 3H). By this time, nerves that received only the LPC injection had also begun to display more highly organized nodal structures with Caspr once again assuming a distinct discrete localization. Kv1.2 was seen to localize to regions adjacent to some of these Caspr-positive distinct regions, and co-localized with Caspr less frequently (Figure 3G), similar to the pattern observed 5d after ES (Figure 3F), albeit less organized.

### ES leads to enhanced macrophage clearance in demyelinated nerves

**Figure 4:**
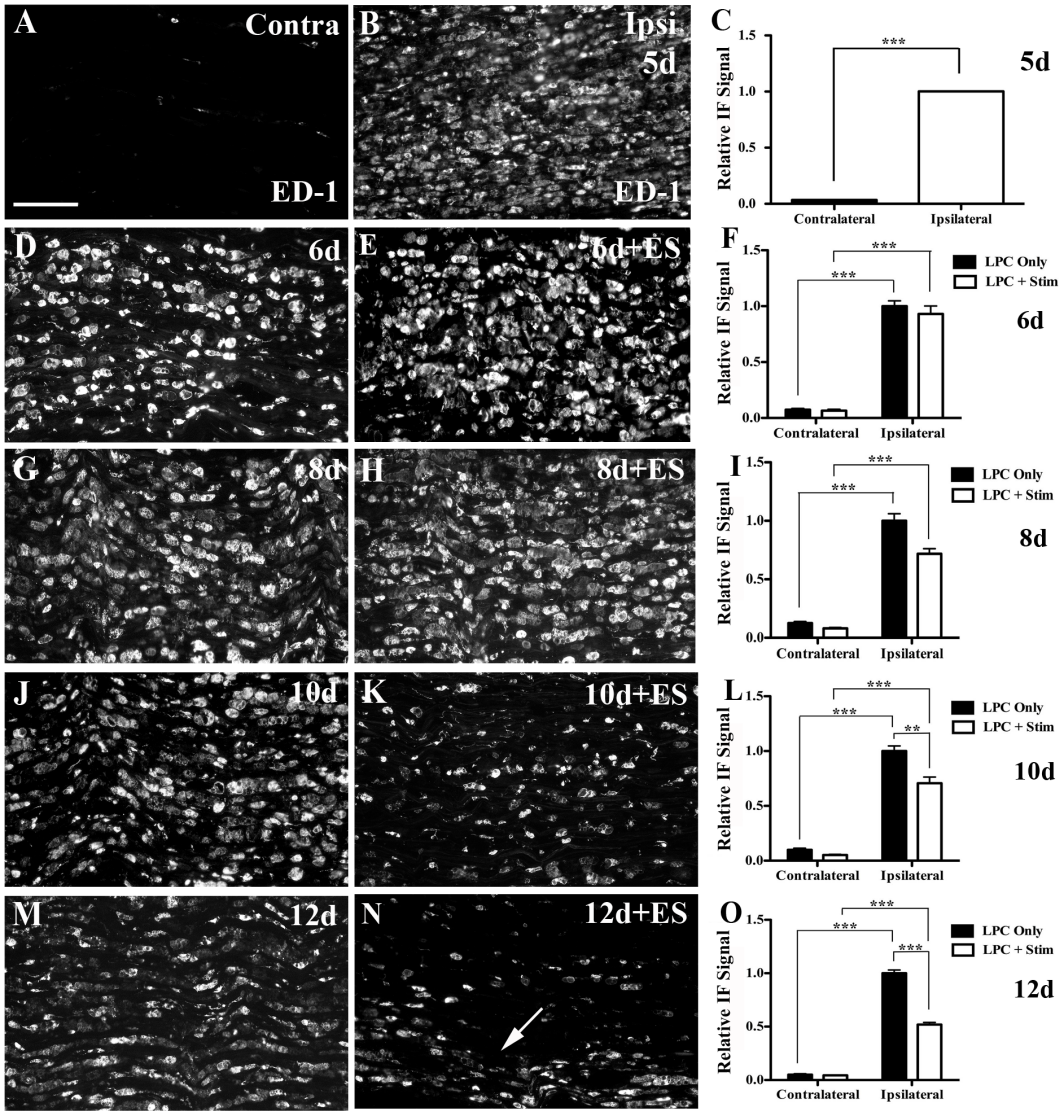
Decrease in number of activated macrophages in demyelinated nerves subjected to 1hr ES. Representative photomicrographs from FG-positive (i.e focally demyelinated) areas of longitudinal tibial nerve sections processed for ED-1 IF to detect activated macrophages. There was marked macrophage infiltration into the demyelination zone 5d post- lysophosphatidyl choline (LPC)/FG injection (B), while contralateral control nerves displayed only minimal ED-1 IF (A). Temporal analysis of LPC focal demyelination +/- ES delivered at 5 d post-LPC as indicated in days post-LPC: 6d (D), 6d+ES (E), 8d (G), 8d+ES (H), 10d (J), 10d+ES (K), 12d (M) or 12d+ES (N) post-LPC. Note: ES results in a significant decrease in the ED-1 IF 10d post-LPC (5d post-ES; K) and 12d post-LPC (7d post-ES; N) relative to LPC only in a pattern suggestive of movement toward lateral edges and egress (arrow). Summary bar graphs of relative changes in IF signal for ED-1 in the zone of demyelination (C, F, I, L, O). Note: for each time point examined, all values were normalized to the mean value of the Average Gray per micron^2^ readings for the nerves ipsilateral to LPC treatment for the LPC Only animal on that slide and for that time point. N = 4-6 animals analyzed per condition; regions quantified per condition: 35 ipsi and 36 contra (5d, LPC only); 89 ipsi and 69 contra (6d, LPC only); 52 ipsi and 53 contra (6d, LPC+Stim); 74 ipsi and 50 contra (8d, LPC only); 66 ipsi and 70 contra (8d, LPC+Stim); 79 ipsi and 64 contra (10d, LPC only); 87 ipsi and 61 contra (10d, LPC+Stim); 122 ipsi and 52 contra (12d, LPC only); 98 ipsi and 71 contra (12d, LPC+Stim). Asterisks indicate significant differences between experimental groups; *P<0.05, ***P<0.001. Scale bar = 100 µm.

Injection of LPC into the tibial branch of the sciatic nerve induces an inflammatory demyelination. At 5d post-LPC injection, numerous macrophages had infiltrated the injection site, demonstrated by intense immunostaining of the macrophage surface marker ED-1 (Figure 4B). The ED-1-positive non-neuronal cells were large, oval shaped cells with a “foamy” appearance, suggestive of active phagocytosis. In contrast, the contralateral (non-injected) sciatic nerve had very little detectable ED-1 (Figure 4A). Within the demyelination zone of both stimulated and non-stimulated nerves there were numerous foamy-appearing macrophages present. This correlated with a significant increase in the amount of the ∼100 kDa ED-1 protein detected via Western blotting for LPC-injected sciatic nerves as compared to both naïve and contralateral (uninjured) sciatic nerves (Figure S4).

At 8d post-LPC injection (three days post-ES), early differences in the levels of ED-1 IF detected in ES and sham stimulated nerves began to emerge. There was a reduction in the amount of detectable ED-1 protein as measured by Western blotting in those nerves receiving one hour brief ES (Figure S4; Figure 4I) albeit they were still higher than levels in uninjured contralateral nerves. By 10d post-LPC injection (five days post-ES) the differences between the stimulated and non-stimulated nerves were visually apparent. While numerous macrophages were still detected in LPC-only injected nerves (Figure 4J), the LPC+ES nerves had significantly fewer (Figure 4K,L). The immunofluorescence findings were confirmed by Western blot analysis (Figure S4). The differences between the two groups were even more apparent and significant at 12d post-LPC (7d post-ES;4M, N, O). Further, there was a shift in the distribution of the activated macrophages within the lesion site from a general scattered one in the non-stimulated nerves, to one localized mainly to the periphery in response to ES, suggestive of immune cell clearance from the endoneurium to the epineurial connective tissue [63], [64]. This suggests that the application of brief ES may enhance clearance of immune cells from the demyelination zone.

In control experiments, nerves from naïve animals that were subjected to one hour ES displayed a very small but expected rise in ED-1 immunoreactivity seven days post-ES, restricted to the site of electrode contact and likely due to the process of surgically exposing the nerve (Figure S2A,B). But this was vastly different from the diffuse robust distribution of macrophages observed in the LPC-injected nerves (Figure 4B).

### Re-appearance and phosphorylation of axonal neurofilaments.is promoted by ES

**Figure 5:**
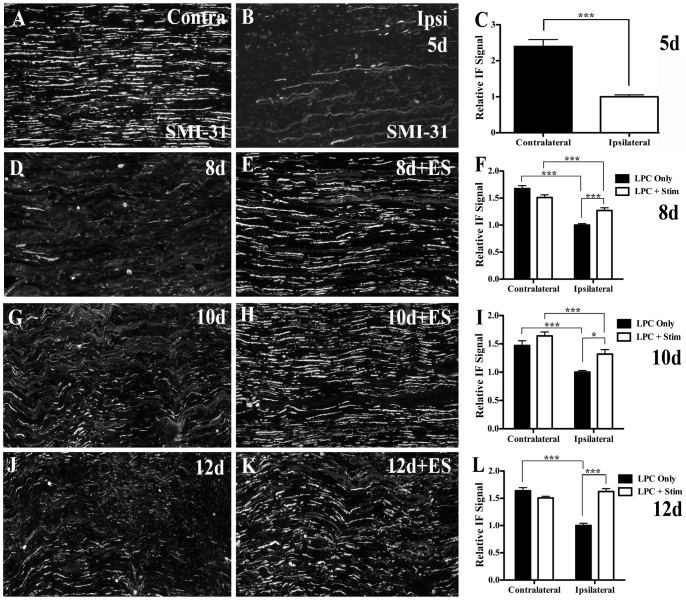
Increased neurofilament phosphorylation in demyelinated nerves subjected to ES. Representative photomicrographs from FG-positive (i.e focally demyelinated) areas of longitudinal tibial nerve sections processed for phosphorylated neurofilament (SMI-31) IF reveal extensive loss of SMI-31 IF (B) as compared to contralateral control nerves which displayed intense SMI-31 immunoreactivity (A). Temporal analysis of LPC focal demyelination +/- ES as indicated in days post-LPC. Note: In the LPC+Stim group there was dramatically increased SMI-31 IF signal apparent as early as 8d post-LPC (3d post-ES; D), which was increasingly stronger in the 10d (5d post-ES; F) and 12d (7d post-ES; H) tissue. In contrast, in the LPC only group there was just a slight increase in SMI-31 IF signal beginning 10d post-LPC. Note: for each time point examined, all values obtained were normalized to the mean value of the Average Gray per micron^2^ readings for the nerves ipsilateral to LPC treatment for the LPC only animal on that slide and for that time point. N = 4-6 animals analyzed per condition; regions quantified per condition: 45 ipsi and 59 contra (5d, LPC only); 57 ipsi and 82 contra (8d, LPC only); 83 ipsi and 72 contra (8d, LPC+Stim); 72 ipsi and 70 contra (10d, LPC only); 83 ipsi and 72 contra (10d, LPC+Stim); 75 ipsi and 82 contra (12d, LPC only); 50 ipsi and 41 contra (12d, LPC+ES). Scale bar = 100 µm.

In naïve control nerves, axons display robust phosphorylated neurofilament expression as detected by SMI-31 IF (Figure 5A). Five days following LPC injection, phosphorylated neurofilament levels were dramatically reduced in the LPC-injected nerves (Figure 5B) suggesting either a loss of neurofilaments and/or neurofilament dephosphorylation in response to the focal demyelination. Remarkably, the LPC-injected nerves that underwent brief ES had recovered most of the lost phosphorylated neurofilament expression as early as 8d post-LPC injection (3d post-ES; Figure 5E), consistent with the observed onset of increased MBP expression and the re-appearance of thinly remyelinated axonal profiles (Figure 2H). This increase in phosphorylated neurofilament expression was not observed in the focally demyelinated nerves not subjected to ES (Figure 5F). The recovery of phosphorylated neurofilament expression in the ES nerves persisted at 10 and 12 d post-LPC (5 and 7 d post-ES respectively; Figure 5H,K). In contrast, non-stimulated nerves only displayed a limited recovery of phosphorylated neurofilament expression beginning 10 days post-LPC (Figure 5G,J).

To confirm the IF observations, Western blot analysis was performed. The levels of total neurofilament (NF) and phosphorylated neurofilament (SMI-31) expression at 5d, 8d and 10d post-LPC with or without ES were compared to naïve and contralateral control nerves (Figure S5). By 5d post-LPC, a dramatic decrease in the level of both total and phosphorylated neurofilament was detected. In protein extracts from stimulated nerves there was an increase in both neurofilament and phosphorylated neurofilament protein levels, though they remained lower than that in both the naïve and contralateral controls (Figure S5). The findings suggest that brief ES promotes the expression of axonal neurofilament proteins and their phosphorylation in the focally demyelinated nerve.

**Figure 6:**
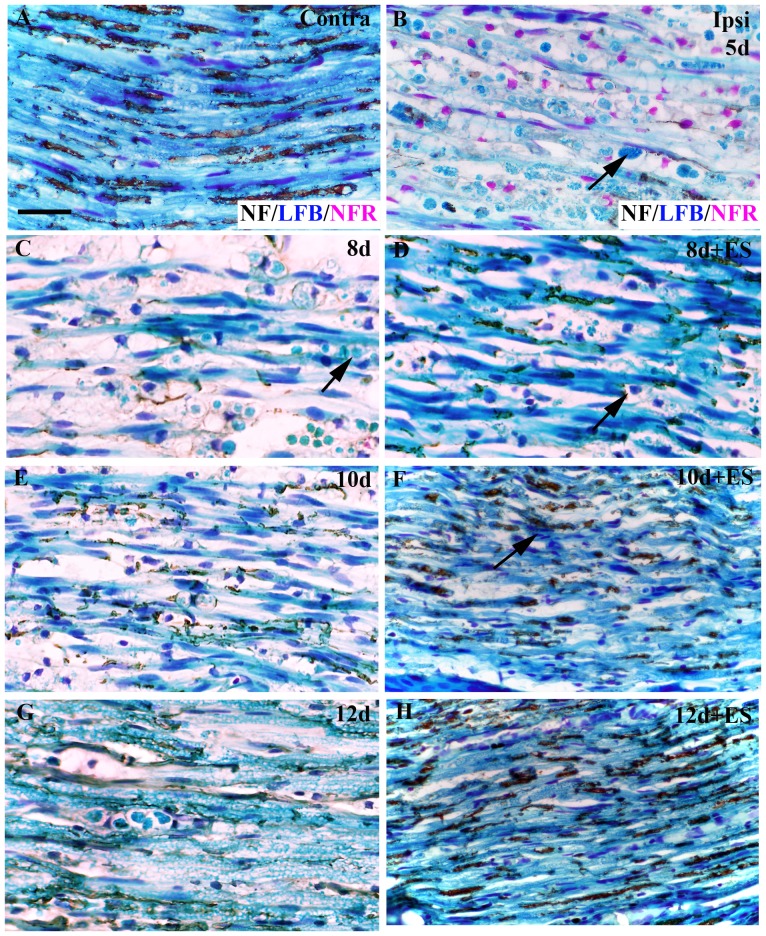
Neurofilament expression increases coincident with reappearance of myelinated axons in demyelinated nerves subjected to ES. Representative photomicrographs from FluoroGold (FG)-positive (i.e.focally demyelinated) areas of longitudinal tibial nerve sections immunostained for neurofilament proteins (NF – brown/black) then stained for the presence of myelin (Luxol Fast Blue [LFB] - blue) and nuclei (nuclear fast red [NFR] – purple/darkblue). Uninjured (*contralateral*) control tibial nerves displayed intense uniform LFB staining, indicative of abundant myelin with prominent linear NF immunostaining (A) and elongated dark blue Schwann cell nuclei. Five days post tibial nerve injection of lysophosphatidyl choline (LPC)/FG, extensive NF and myelin loss is observed coupled with infiltration by numerous macrophages filled with largely unprocessed myelin debris (B,C arrow). Temporal analysis of LPC-focally demyelinated nerves +/- ES as indicated in days post-LPC. Note: In the LPC+ES group there was increasing linear NF immunoreactivity detected (F,H) with higher levels of uniform LFB staining consistent with myelinated axons, and the appearance of macrophages devoid of myelin debris apparent as early as 8d post-LPC (3d post-ES; D, arrow). The LFB staining was even stronger in the 10d (5d post-ES) animals where presumptive Schwann cells now display elongated nuclei (F, arrow) and 12d (7d post-ES; H) post-ES tissue. In contrast, in the LPC only group there was just a slight increase in NF immunoreactivity beginning 10d post-LPC (E), but it was much less robust than that observed in the stimulated as well as the contralateral control nerves. Notably in the nonstimulated nerves the immune cell infiltration is still largely unresolved at the 12d post-LPC time point. Scale bar = 5 µm.

We also used classic histologic approaches to examine the overall impact of ES on LPC demyelinated nerves. To do this, sections were processed for neurofilament immunohistochemistry then stained with classic histological stains to detect presumptive macrophages and myelin. In naïve control nerves immunoreactivity for neurofilament proteins was readily visible (Figure 6A) and assumed a linear pattern. This was also intense Luxol Fast Blue staining, as one would expect with normal myelin content, similar to the results obtained by MBP IF (Figure 2A). Numerous elongated nuclei (consistent with Schwann cell nuclei) were observed in close association with the myelin stain and neurofilaments. In sharp contrast, at 5d post-LPC injection, an extensive infiltration of macrophages had occurred consistent with the ED-1 IF results (Figure 4). These cells were foamy in appearance and contained myelin debris (Figure 6B). There was also a distinct loss of neurofilament, suggesting that the focal demyelination had indeed impacted neurofilament expression. Despite this, the axons appeared to be structurally intact based on the robust linear β-III tubulin IF staining observed (Figure 1D).

Eight days post-LPC (three days post-ES), the non-stimulated nerves still had dramatically reduced myelin levels, a continued presence of myelin debris-containing macrophages and largely lacked detectable neurofilament immunostaining (Figure 6C). In contrast, the ES nerves displayed a higher level of neurofilament expression,,consistent with the SMI-31 IF and Western blot data (Figures 5D, 6D; Figure S5). In addition, thin segments of myelin were detected in close association with the linear neurofilament immunoreactive signal, suggestive of early remyelination (Figure 6D). While the stimulated nerves still had numerous macrophages (many containing myelin debris), there was also an emergence of some smaller less foamy macrophages largely devoid of visible myelin (Figure 6D). Similar results were observed at 10 (Figure 6E,F) and 12 d (Figure 6G,H) post-LPC (5 and 7 d post-ES, respectively). The nerves not receiving ES displayed only minimal recovery of neurofilament immunoreactivity at 10d post-LPC, remaining largely demyelinated with numerous foamy macrophages still present (Figure 6E,G). In contrast, the stimulated nerves displayed higher levels of detectable neurofilaments with myelinated axon profiles more evident and elongated nuclei resembling those of Schwann cells aligned with the neurofilaments (Figure 6F, arrow). The stimulated nerves were also largely devoid of macrophages by 12d post-LPC, consistent with the ED-1 IF findings (Figure 2N). They more closely resembled the contralateral control nerves, albeit with a myelin density that was still less than that observed in the contralateral control nerves (Figure 6H). By 12d post-LPC the nerves that did not receive brief ES displayed increased neurofilament immunoreactivity (Figure 6G), similar to that observed in the ES nerves at eight days post-LPC. At this time point the non-stimulated nerves also showed the first indication that the inflammatory process was resolving, by the presence of non-myelin containing macrophages. Again, Western blot analysis confirmed these observations (Figure S4; Figure S5).

Taken together, the above results suggest that brief ES promotes not only the re-appearance of the neurofilament proteins, but also promotes a more rapid return to an axon-protective phosphorylated state amenable to remyelination.

### Gradual attenuation of the Schwann cell reactive state following brief ES

**Figure 7:**
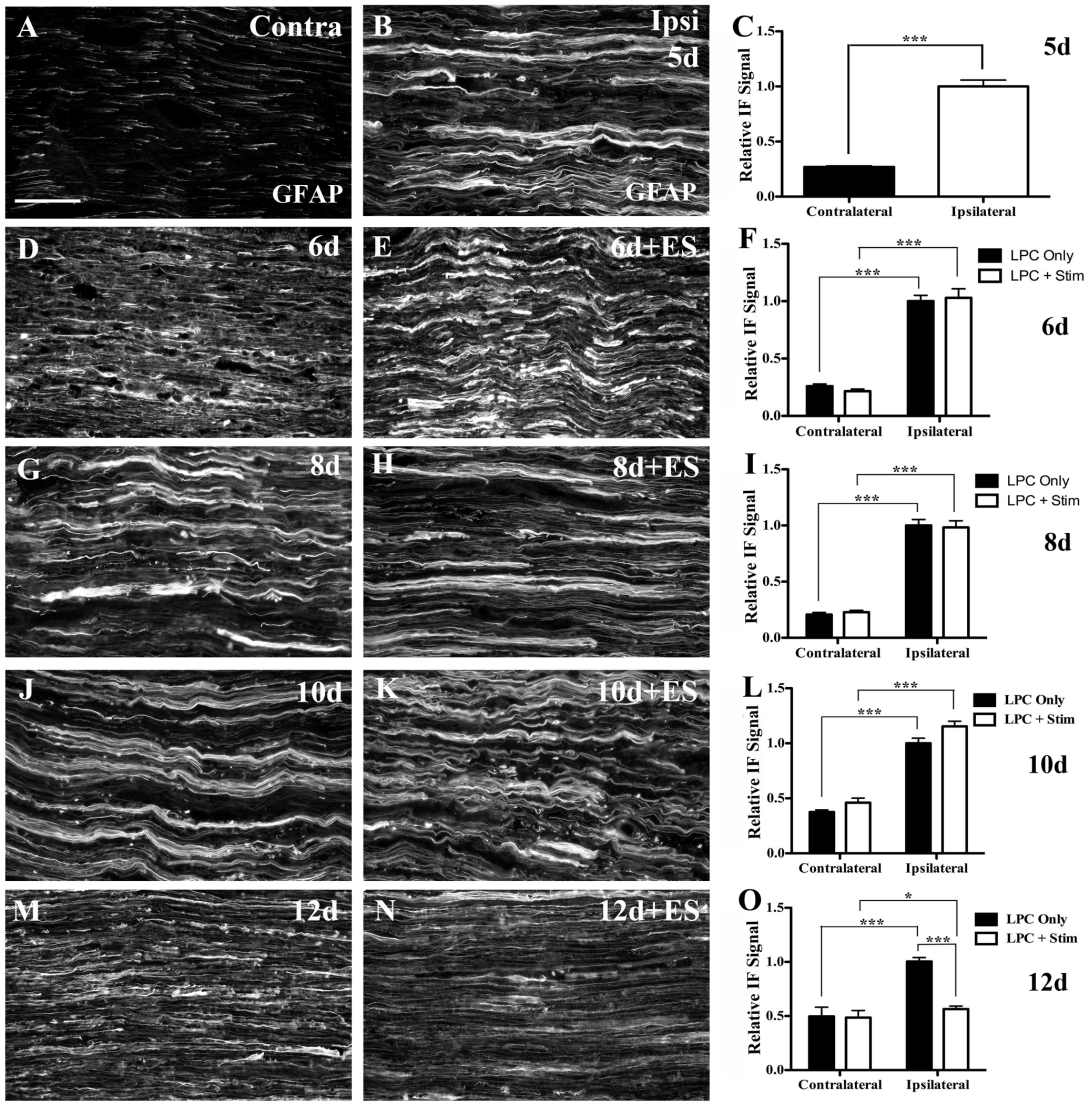
Decreased reactive gliosis following ES delivered 5d post-demyelination. Representative immunofluorescence photomicrographs from FluoroGold (FG) positive areas of tibial nerve sections processed for glial fibrillary acidic protein (GFAP) immunofluorescence (IF) to detect reactive Schwann cells. Contralateral intact control nerves displayed only minimal GFAP IF (A). However 5d post-lysophosphatidyl choline (LPC)/FG injection there was a marked increase in GFAP IF (B). Temporal analysis of LPC focal demyelination +/- ES as indicated in days post-LPC: 6d (D), 6d+ES (E), 8d (G), 8d+ES (H), 10d (J) 10d+ES or 12d (M) 12d+ES (N) post-LPC. Note: ES resulted in a significant decrease in the GFAP IF signal detected at 12d post-LPC (7 days post-ES; N) relative to 12d LPC only (M). Summary bar graphs of relative changes in immunofluorescence signal for GFAP from sections of tibial nerve bridging the site of demyelination (C, F, I, L, O). N = 4-6 animals analyzed per condition; regions quantified per condition: 49 ipsi and 32 contra (5d, LPC only); 73 ipsi and 64 contra (6d, LPC only); 60 ipsi and 48 contra (6d, LPC+Stim); 84 ipsi and 68 contra (8d, LPC only); 53 ipsi and 60 contra (8d, LPC+Stim); 89 ipsi and 74 contra (10d, LPC only); 86 ipsi and 76 contra (10d, LPC+Stim); 142 ipsi and 74 contra (12d, LPC only); 108 ipsi and 80 contra (12d, LPC+Stim). Asterisks indicate significant differences between experimental groups; *P<0.05, ***P<0.001. Scale bar  =  100 µm.

Focal demyelination was also associated with an increased reactive state in Schwann cells (SC), as evidenced by increased IF signal for the cytoskeletal protein glial fibrillary acidic protein (GFAP) in the LPC-injected tibial nerves (Figure 7B). GFAP IF was significantly higher than that observed in normal (non-injected) control nerve (Figure 7A, B, C). Five days following brief ES there was no significant difference in the GFAP levels detected between the stimulated nerves and their non-stimulated counterparts in the zone of demyelination. Both displayed elevated GFAP expression indicative of robust SC activation (Figure 7D-L).

However by 12d post-LPC injection (7d post-ES) a marked difference between the animals that received the brief ES and those that did not had emerged. The LPC-only animals still expressed high levels of GFAP (Figure 7M). However, in ES nerves the GFAP IF levels were now significantly below that observed in the LPC-only animals (Figure 7N, O). This suggests that ES gradually results in a more rapid resolution of the activated state of SCs in the demyelination zone.

Similar to the observed for ED-1, ES alone was not sufficient to induce a reactive state in SCs within naïve nerves seven days post-ES (Figure S3A, B).

### ES increases BDNF levels in focal demyelination zone

**Figure 8:**
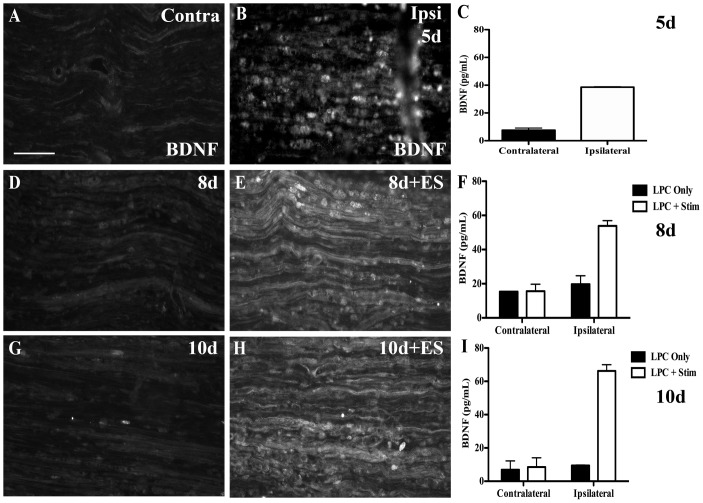
Increased BDNF protein in demyelination zone following 1 hr ES. Representative immunofluorescence (IF) photomicrographs from FG-positive focally demyelinated areas of tibial nerve sections immunostained for BDNF. Contralateral control nerves displayed only minimal BDNF IF (A). There was a marked increase in BDNF observed 5d post- lysophosphatidyl choline (LPC)/FG injection (B), largely within cells identified as ED-1 positive macrophages (data not shown). Temporal analysis of LPC focal demyelination +/- ES delivered 5 d post_LPC and as indicated in days post-LPC: 8d (D), 8d+ES (E), 10d (G), 10d+ES (H). Note: ES results in an increase in the BDNF IF signal detected 8 and 10 d post-LPC (3 and 5 days post-ES; E,H) relative to LPC only nerves (D,G). Summary bar graphs of BDNF protein levels measured by ELISA in samples of sciatic nerve bridging the site of demyelination are in agreement with BDNF IF (C, F, I). Asterisks indicate significant differences between experimental groups; **P<0.01. Scale bar = 100 µm.

It has been previously reported that BDNF plays a key role in peripheral nerve myelination [34], [35] and that in models of axonal transection and repair, brief ES results in increased BDNF expression that is presumably be released from growing axon tips [48], [53], [54], [55]. However, in our model of focal demyelination, the axons are still intact. Thus, we sought to determine if brief ES could affect an increase in BDNF at the site of focal demyelination. Using BDNF immunohistochemistry and ELISA analysis, a persistent low level of BDNF was detected in control sciatic nerves (Figure 8A). Five days post-LPC increased BDNF levels were detected within the demyelination zone, localizing predominantly to cells identified as ED-1-positive macrophages (Figure 8B, colocalization data not shown). BDNF ELISA revealed that the BDNF content in the demyelination zone 5 days post-LPC injection was 38.6 +/-0.3 pg/ml (s.e.m.), as compared to 7.6 +/- 1.6 pg/ml (s.e.m.) in an equivalent region of the contralateral nerve (Figure 8C). Eight days post-LPC injection (3d post-ES) the ES nerves contained even higher levels of BDNF at 53.8 +/- 3.1 pg/ml (s.e.m.), as compared to the LPC-only nerves, which had declined relative to the 5 day post-LPC levels to 19.7 +/- 4.9 pg/ml (s.e.m.). Notably, at this time point the increased BDNF IF signal colocalized with the axonal marker βIII-tubulin, the Schwann cell marker GFAP and the macrophage marker ED-1 (colocalization data not shown; Figure 8D, E, F). This suggests there are additional sources of BDNF in the stimulated nerve not observed in the non-stimulated nerves. The levels of BDNF in the demyelination zone were still elevated in the ES-treated nerves 10d post-LPC (five days post-ES). Nerves receiving brief ES had greater BDNF IF signal and mean BDNF content (66.3 +/- 3.7 pg/ml, s.e.m.), compared to the non-stimulated LPC-treated nerves that had declined to almost the baseline levels of control contralateral nerves (9.5 +/- 0.3 pg/ml, s.e.m.). Once again, there was agreement between the BDNF ELISA and IF data, with the latter appearing similar to that observed in the 8d post-LPC group. The immature pro-BDNF protein has a molecular weight of ∼37 kDa, while the processed, mature peptide has a molecular weight of ∼14 kDA [65]. Analysis of protein extracts from experimental sciatic nerve samples by non-reducing Western blot revealed that BDNF was detected at a molecular weight of ∼25 kDa in both naïve and contralateral control nerves consistent with the predicted weight of a homodimer of the mature BDNF protein. Alterations in the amount of BDNF detected by Western blot (data not shown) validated the IF and ELISA data.

## Discussion

In this study the *in vivo* therapeutic potential of brief electrical axonal stimulation on remyelination of focally demyelinated nerves was examined for the first time. The data identifies ES as a novel strategy to promote remyelination in demyelinating peripheral nerve pathologies and provide insights into the potential underlying mechanisms and cellular events associated with its beneficial effects. The focally demyelinated nerves that received a single bout of electrical stimulation (ES) remyelinated more rapidly relative to nerves that were demyelinated but not stimulated. The cellular events associated with the ES-enhanced remyelination included: elevated levels of BDNF, a promyelinating molecule; axonal protection in the form of re-appearance of neurofilaments in an axon-protective phosphorylated state; increased remyelination as evidenced by increased expression of myelin basic protein (MBP) and supported by increased Luxol Fast Blue staining; accelerated reestablishment of node of Ranvier architecture; enhanced clearance of myelin debris and activated macrophages; and a more rapid return of the Schwann cells to a non-activated state within the demyelination zone.

### Remyelination of demyelinated axons can be accelerated by ES

In the LPC peripheral nerve model of focal nerve demyelination, axons are demyelinated within six days, with the first appearance of thinly remyelinated fibers evident only around 14d post-LPC injection [59]. We found that the ES-promoted remyelination was apparent as early as 3d post-ES (8d post-LPC injection) and clearly evident by 7d post-ES (12d post-LPC). The ES effect on myelination was likely the result of increased neuronal activity, as it was not observed with sham ES, or after action potential blockade at the time of stimulation.

Enhanced neuronal activity has been linked to myelination of both the central and peripheral axons [49], [66], [67], [68]. Our findings are consistent with the ability of ES to promote remyelination in another peripheral nerve pathology, namely regenerating peripheral neurons [54], [69]. While the same stimulation parameters were employed in the present study as in the two regeneration studies (a single bout of ES for 1 hr at 20 Hz), our overall paradigm differed in that the ES was delivered in a delayed fashion once the focal demyelination had occurred (5d post-LPC), as opposed to immediately at the time of nerve injury. This supports that with respect to myelination of axons, even delayed nerve stimulation may be beneficial and reparative. It is unlikely that a single set of ES parameters will result in favorable outcomes for all pathologies as regional differences in the response to ES have been noted [68]. The current study also does not give insight as to whether ES therapy is able to mitigate the demyelinating insult if administered at the same time as the demyelinating agent, such as was seen for methylprednisolone in LPC-induced demyelination of central axons [70]. Finally, the invasive nature of the current paradigm makes it unlikely to be employed in the same manner as in this study. Instead alternative strategies to enhance neuronal activity such as specific motor exercises will have to be tested [71], [72].

### BDNF and myelination

The link between brief ES and remyelination in injury models may be attributable to the ES-induced elevations in neuronal BDNF observed. In the previous regeneration studies this extra BDNF would presumably be released from the growing axon tips [54], [69], [73] with one study implicating BDNF in the remyelination of regenerated axons [69] and another demonstrating the contribution of macrophage-derived BDNF to axonal regeneration [74]. The present study revealed an ES-induced sustained increase in BDNF levels most apparent in axons within the demyelination zone, at times coincident with the onset of remyelination that lasted as long as the last time point examined (10d post-LPC; Figure 8). It is conceivable that this BDNF could be released directly from the focally demyelinated axons and/or by the infiltrating macrophages [75]. In sensory neurons BDNF is normally co-packaged with neuropeptide transmitters in large dense core vesicles [76] which have been shown to be released from both the unmyelinated axons, as well as at the axon terminals [77], [78]. It is also conceivable that the ES-associated elevated axonal- or macrophage-derived BDNF in the present study is causally linked to the enhanced remyelination. In support of this, previous studies that either modulate BDNF availability or in the absence of endogenous BDNF utilize small trkB agonists have shown BDNF or activation of its receptor to be critically linked to effective myelination [43], [79], [80].

While ES-associated increases in MBP or LFB staining are not definitive proof that remyelination has occurred, when taken together with the observed recapitulation of a discrete distribution of the paranodal and juxtaparanodal markers, Caspr and Kv1.2 as part of essential nodal architecture, they support that remyelination has most likely occurred. Demyelinated axons no longer have the axon-Schwann cell interactions critical for the clustering of nodal, paranodal and juxtaparanodal proteins [21], [22] thus, the normally highly localized pattern of nodal, paranodal and juxtaparanodal proteins takes on a more diffuse appearance consistent with what we observed 5d post-LPC. It has been shown that the highly localized appearance of Caspr is one of the first indications axons are undergoing myelination [81] and the re-appearance of this restricted staining pattern, along with quantitative differences in the number of visible nodes per field of view at earlier time points in the nerves treated with delayed brief ES support that remyelination had indeed taken place. Interestingly, BDNF has also been shown to promote the clustering of Caspr and sodium channels in oligodendrocyte progenitor cell/sensory neuron co-cultures [82].

### Brief ES promotes axon-protective neurofilament phosphorylation

In addition to promoting early remyelination of axons, brief ES of demyelinated nerve promoted the re-appearance of phosphorylated axonal neurofilaments, a key determinant of axonal health and capacity to be remyelinated. Maintaining electrical activity factors greatly into preserving axon health, with a lack of neurofilament proteins being associated with reduced sciatic nerve conduction velocity [10]. Neurofilament proteins play a key role in the determination of axon caliber [8], [9], which along with electrical activity [83] are important factors in determining whether an axon will be myelinated [84]. As it is the phosphorylation state that appears to be responsible for protection from proteolysis and maintaining axon diameter [9], [14], [15], a more rapid return to this state is likely beneficial for preservation of both axonal health and perhaps more importantly, axon number. Axonal degeneration is widely observed in demyelinating neuropathies in which decreased neurofilament number as well as decreased phosphorylation has been noted [20], [85], [86] consistent with what we observed. In our study, a return to a state in which neurofilaments have reappeared and are in the protective phosphorylated state was evident at the first time point post-ES examined (8d post-LPC; 3d post-ES) and coincided with the onset of the observed remyelination and reorganization of the paranodal and juxtaparanodal regions. Whether the ES increases in BDNF are linked to the neurofilament phosphorylation is not known, but has been shown for cortical neurons [87].

The increased NF and SMI-31 immunoreactivity in the stimulated nerves remained present throughout all time points examined, revealing a tremendous impact of a single bout of ES on this axis. Nerves not subjected to brief ES also began to display a slight increase in NF and SMI-31 immunoreactivity, but this was not apparent until later time points (10-12 d post-LPC) and was much less robust than that of the ES nerves. These results suggest that the stimulation procedure is successfully protecting the axons by promoting their return to a competent state amenable to myelination. Communication between competent axons and their associated Schwann cells is essential for the initiation of the myelination program and for ongoing maintenance of the myelin sheath [88], [89], [90]. Thus, in addition to promoting axonal survival, it is possible that ES is also enhancing the communication between the axons and the associated Schwann cells in order to affect a more rapid repair process.

### Demyelination-associated immune responses resolve more quickly with ES

Beyond enhancing remyelination, ES also attenuated and/or accelerated the inflammatory immune response that accompanies the generation and resolution of these demyelinating insults. In electrically stimulated animals, beginning at 10d post-LPC (5d post-ES), macrophages that had infiltrated the lesion site transitioned from being distributed throughout the nerve to being concentrated near the periphery, with many macrophages now devoid of myelin debris. It is known that the majority of macrophages responsible for the phagocytosis of myelin debris following a demyelinating insult are derived from the peripheral circulation, with the resident macrophages of the peripheral nerve making only a minor contribution [91]. Once the process of phagocytosis has been completed, these peripheral blood-derived macrophages may undergo apoptosis at the lesion site [63] or exit the lesion site and eventually return to the circulation via the SC basal lamina [63], [64]. Because at earlier time points (6d and 8d post-LPC) both stimulated and non-stimulated nerves displayed similar levels and distribution of ED-1 positive cells, consistent with that observed in the histologically processed sections, the differences observed between the two groups beginning 10d post-injection (5d post-ES) were unlikely the result of impairment in the initial infiltration of the nerve by the peripheral blood-derived macrophages. Rather our findings are consistent with an ES-enhanced resolution of inflammation associated with the focal demyelination. This supports the idea that brief ES heightens macrophage activity, as those animals receiving brief ES displayed signs consistent with macrophage egress sooner than their non-stimulated counterparts. This could be due to either increased metabolic activity of the macrophages and/or, upregulation of a signaling pathway that effects early clearance of the phagocytic cells from the lesion site. While the signals that direct this cellular egress are not fully understood, evidence implicates the Nogo family of receptors (NgRs). Macrophages express NgRs on their surface that upon interaction with ligands (such as MAG) present in myelin are capable of initiating repulsive migration [92]. ES may affect expression of these receptors and/or their ligands, which might underlie the observed enhanced clearance of macrophages and is the target of future studies.

Finally, our observed attenuation of a reactive state for Schwann cells within the lesion site was only evident at the last time point examined 12d post-LPC (7d post-ES). This may be linked to the egress/resolution of the activated macrophages in affected zone, as they have been shown to contribute to the reactive state of Schwann cells [64]. In uninjured nerve, GFAP expression is largely restricted to the population of non-myelinating Schwann cells. However, upon injury there is a rapid increase in GFAP immunoreactivity that also includes the myelin forming population of Schwann cells distal to the lesion site [93], [94]. Perhaps the ability of brief ES to attenuate GFAP expression in the demyelination zone is linked to a switch in the dedifferentiated Schwann cell back to a myelinating phenotype.

### Summary and implications

Conventional therapeutic approaches for the treatment of demyelinating disorders tend to focus on modulation of the immune response believed responsible for the generation of the demyelinated lesions [95], [96]. While this may help to the reduce relapse rate and delay progression of the disorder, immune system modulation does not tackle the fundamental problem of remyelination of these damaged areas of the nervous system and preservation of the demyelinated axon.

The current study revealed brief stimulation of focally demyelinated nerves has a tremendous impact on many of the cellular determinants associated with axon protection and effective remyelination. Whether these are all causally linked to the observed increases in BDNF expression is the focus of ongoing research. Regardless, these results are encouraging as they indicate that it is indeed possible to significantly enhance the existing intrinsic remyelination processes in the peripheral nervous system *in vivo* by increasing neuronal activity, giving hope for the more debilitating demyelinating disorders of the central nervous system.

## Supporting Information

Figure S1 **Increased neuronal activity effects increases in myelin basic protein (MBP) observed following electrical stimulation of focally demyelinated nerve.** Representative immunofluorescence photomicrographs of tibial branch of sciatic nerve sections immunostained for MBP. Naïve (uninjured) nerves displayed intense MBP immunoreactivity (A). Electrical stimulation (ES) of uninjured nerves appeared to have no affect on the integrity of the myelin sheath (B). Sham ES and blockage of action potential conduction through local application of lidocaine did not result in the increased remyelination following unilateral focal LPC demyelination normally observed upon application of ES (C, D). This indicates that the increased MBP immunoreactivity observed in electrically stimulated nerves is related to the axonal activity induced by the ES procedure. Scale bar = 100 µm.
(TIF)

Figure S2 **Increased neuronal activity is required to effect reductions in activated macrophage (ED1) immunoreactivity in focally demyelinated regions.** Representative immunofluorescence photomicrographs of tibial nerve sections immunostained for ED1. Naïve (uninjured) nerves displayed minimal ED1 immunoreactivity (A). Electrical stimulation (ES) alone did not trigger an inflammatory response nor an infiltration of activated macrophages into the stimulation site (B). Sham stimulation and blockade of action potential conduction through local application of lidocaine did not result in an increase in macrophage clearance from the lesion site (C, D). This indicates that the enhanced clearance of macrophages from the demyelinated lesions of animals receiving electrical stimulation (ES) is related to the activity induced by the ES procedure. Scale bar = 100 µm.
(TIF)

Figure S3 **Increased neuronal activity is required to effect a reduction in Schwann cell reactivity in zones of focal demyelination.** Representative immunofluorescence photomicrographs (20x magnification) of sciatic nerve sections immunostained for glial fibrillary acidic protein (GFAP). Naïve (uninjured) nerves display some GFAP immunoreactivity, reflecting the population of non-myelinating Schwann cells present within peripheral nerves (A). Electrical stimulation alone did not induce reactive gliosis (B). Sham stimulation and blockage of action potential conduction through local application of lidocaine did not result in a decrease in reactive gliosis within the lesion site (C, D). This indicates that the reduction in Schwann cell reactivity observed within the demyelinated lesions of animals receiving electrical stimulation is related to the activity induced by the electrical stimulation procedure. Scale bar = 100 µm.
(TIF)

Figure S4 **1hr electrical stimulation (ES) 5 days post-lysophosphatidyl choline (LPC) results in decreased ED-1 content beginning 8d post-LPC.** Representative Western blots of sciatic nerve extract probed for ED-1. Immunoblots were run in duplicate from pooled nerve samples from 3 animals/experimental condition. Densitometry readings were normalized to the loading control β-III tubulin within each lane and compared to the mean densitometry reading of the two lanes of naïve sciatic nerve protein extract run alongside the demyelinated nerve extracts in each gel. There was a marked increase in detectable ED-1 observed 5d post-lysophosphatidyl choline (LPC) injection into the tibial branch of the sciatic nerve, as compared to that observed in protein extract from both naïve and contralateral (uninjured) nerves. As early as three days post-ES (8d post-LPC), there was a decrease in the amount of detectable ED-1 in the stimulated nerves. This drop preceded the visual differences observed immunohistochemically (Figure 4J, K). Levels of detectable ED-1 showed further decline 5d post-ES (10d post-LPC), where they reached levels not significantly different than that of the naïve or contralateral (uninjured) controls. Asterisks indicate significant differences between experimental groups; *P<0.05, **P<0.01, ***P<0.001, Student's t-test.
(TIF)

Figure S5 **1hr brief electrical stimulation (ES) results in increased expression of total and phosphorylated neurofilament proteins.** Representative Western blot of tibial nerve demyelination zone protein extract and probed for total (NF) and phosphorylated neurofilaments (SMI-31). Immunoblots were run in duplicate from pooled nerve samples from 3 animals/experimental condition. Densitometry readings were normalized to the loading control β-III tubulin within each lane and compared to the mean densitometry reading of the two lanes of naïve sciatic nerve protein extract run alongside the demyelinated nerve extracts in each gel. Naïve and contralateral control nerves displayed intense NF and SMI-31 immunoreactivity. There was a marked decrease in both NF and SMI-31 band intensity observed 5d post-lysophosphatidyl choline (LPC)/FG injection into the tibial branch of the sciatic nerve. ES resulted in an increase in the amount of detectable NF and SMI-31 proteins 3 and 5 days post-ES (8 and 10 days post-LPC), consistent with the immunohistochemical observations and quantification (see Figure 5). In focally demyelinated nerves that did not undergo ES the levels of NF and SMI-31 remained low. Asterisks indicate significant differences between experimental groups; *P<0.05, **P<0.01, ***P<0.001, Student's t-test.
(TIF)

## References

[1] Steinman L (1996) Multiple sclerosis: A coordinated immunological attack against myelin in the central nervous system. Cell 85: 299–302. 8616884 10.1016/s0092-8674(00)81107-1

[2] Winer JB, Hughes AC, Osmond C (1988) A prospective study of acute idiopathic neuropathy. I. Clinical features and their prognostic value. Journal of Neurology, Neurosurgery and Psychiatry 51: 605–612. 10.1136/jnnp.51.5.605 PMC1033062 2841422

[3] Yuki N, Hartung H-P (2012) Guillain-Barre syndrome. The New England Journal of Medicine 366: 2294–2304. 22694000 10.1056/NEJMra1114525

[4] Chio A, Cocito D, Leone M, Giordana MT, Mora G, et al (2003) Guillain-Barre syndrome a prospective, population-based incidence and outcome survey. Neurology 60: 1146–1150. 12682322 10.1212/01.wnl.0000055091.96905.d0

[5] Hughes RAC, Cornblath DR (2005) Guillain Barre syndrome. Lancet 366: 1653–1656. 16271648 10.1016/S0140-6736(05)67665-9

[6] Silber E, Sharief MK (1999) Axonal degeneration in the pathogenesis of multiple sclerosis. Journal of the Neurological Sciences 170: 11–18. 10540030 10.1016/s0022-510x(99)00178-1

[7] Drenthen J, Jacobs BC, Maathuis EM, van Doorn PA, Visser GH, et al (2013) Residual fatigue in Guillain-Barre dyndrome is related to axonal loss. Neurology 81: 1827–1831. 24163277 10.1212/01.wnl.0000436073.21406.e6

[8] Friede RL, Samorajski T (1970) Axon caliber related to neurofilaments and microtubules in sciatic nerve fibers of rats and mice. Anatomical Record 167: 379–388. 5454590 10.1002/ar.1091670402

[9] Hoffman PN, Cleveland DW, Griffin JW, Landes PW, Cowan NJ, et al (1987) Neurofilament gene expression: A major determinant of axonal caliber. Proceedings of the National Academy of Sciences USA 84: 3472–3476. 10.1073/pnas.84.10.3472 PMC304893 3472217

[10] Sakaguchi T, Okada M, Kitamura T, Kawasaki K (1993) Reduced diameter and conduction velocity of myelinated fibers in the sciatic nerve of a nerofilament-deficient mutant quail. Neuroscience Letters 153: 65–68. 8510825 10.1016/0304-3940(93)90078-y

[11] Lee VM, Carden MJ, Schlaepfer WW, Trojanowski JQ (1987) Monoclonal antibodies distinguish several differentially phosphorylated states of the two largest rat neurofilament subunits (NF-H and NF-M) and demonstrate their existence in the normal nervous system of adult rats. The Journal of Neuroscience 7: 3474–3488. 3119789 10.1523/JNEUROSCI.07-11-03474.1987 PMC6569035

[12] Pant HC, Veeranna TK (1995) Neurofilament phosphorylation. Biochemistry and Cell Biology 73: 575–592. 8714676 10.1139/o95-063

[13] Michailov GV, Sereda MW, Brinkmann BG, Fischer TM, Haug B, et al (2004) Axonal neuregulin-1 regulates myelin sheath thickness. Science 304: 700–703. 15044753 10.1126/science.1095862

[14] Goldstein ME, Sternberger NH, Sternberger LA (1987) Phosphorylation protects neurofilaments against proteolysis. Journal of Neuroimmunology 14: 149–160. 3029175 10.1016/0165-5728(87)90049-x

[15] Greenwood JA, Troncoso JC, Costello AC, Johnson GVW (1993) Phosphorylation modulates calpain-mediated proteolysis and calmodulin binding of the 200-kDA and 160-kDa neurofilament proteins. Journal of Neurochemistry 61: 191–199. 8515266 10.1111/j.1471-4159.1993.tb03555.x

[16] Kamakura K, Ishiura S, Sugita H, Toyokura Y (1983) Identification of Ca2+-activated neutral protease in the peripheral nerve and its effects on neurofilament degeneration. Journal of Neurochemistry 40: 908–913. 6300327 10.1111/j.1471-4159.1983.tb08072.x

[17] Pant HC (1988) Dephosphorylation of neurofilament proteins enhances their susceptibility to degradation by calpain. Journal of Biochemistry 256: 665–668. 10.1042/bj2560665 PMC1135461 2851997

[18] Starr R, Attema B, DeVries GH, Monteiro MJ (1996) Neurofilament phosphorylation is modulated by myelination. Journal of Neuroscience Research 44: 328–337. 8739151 10.1002/(SICI)1097-4547(19960515)44:4<328::AID-JNR3>3.0.CO;2-E

[19] de Waegh SM, Lee VM-Y, Brady ST (1992) Local modulation of neurofilament phosphorylation, axonal caliber, and slow axonal transport by myelinating Schwann cells. Cell 68: 451–463. 1371237 10.1016/0092-8674(92)90183-d

[20] Trapp BD, Peterson J, Ransohoff RM, Rudick R, Mork S, et al (1998) Axonal transection in the lesions of multiple sclerosis. The New England Journal of Medicine 338: 278–285. 9445407 10.1056/NEJM199801293380502

[21] Arroyo EJ, Sirkowski EE, Chitale R, Scherer SS (2004) Acute demyelination disrupts the molecular organization of peripheral nervous system nodes. The Journal of Comparative Neurology 479: 424–434. 15514980 10.1002/cne.20321

[22] Karimi-Abdolrezaee S, Eftekharpour E, Fehlings MG (2004) Temporal and spatial patters of Kv1.1 and Kv1.2 protein and gene expression in spinal cord white matter after acute and chronic spinal cord injury in rats: implications for axonal pathophysiology after neurotrauma. European Journal of Neuroscience 19: 577–589. 14984408 10.1111/j.0953-816x.2004.03164.x

[23] Rasband MN, Trimmer JS, Schwarz TL, Levinson SR, Ellisman MH, et al (1998) Potassium channel distribution, clustering, and function in remyelinating rat axons. The Journal of Neuroscience 18: 36–47. 9412484 10.1523/JNEUROSCI.18-01-00036.1998 PMC6793423

[24] Plemel JR, Manesh SB, Sparling JS, Tetzlaff W (2013) Myelin inhibits oligodendroglial maturation and regulates oligodendrocytic transcription factor expression. Glia 61: 1471–1487. 23839973 10.1002/glia.22535

[25] Ruckh JM, Zhao JW, Shadrach JL, van Wijngaarden P, Rao TN, et al (2012) Rejuvenation of regeneration in the aging central nervous system. Cell stem cell 10: 96–103. 22226359 10.1016/j.stem.2011.11.019 PMC3714794

[26] Kotter MR (2006) Myelin impairs CNS remyelination by inhibiting oligodendrocyte precursor cell differentiation. The Journal of Neuroscience 26: 328–332. 16399703 10.1523/JNEUROSCI.2615-05.2006 PMC6674302

[27] Bruck W (1997) The role of macrophages in Wallerian degeneration. Brain Pathology 7: 741–752. 9161725 10.1111/j.1750-3639.1997.tb01060.x PMC8098515

[28] Griffin JW, George R, Lobato C, Tyor WR, Yan LC, et al (1992) Macrophage responses and myelin clearance during Wallerian degeneration: Relevance to immune-mediated demyelination. Journal of Neuroimmunology 40: 153–166. 1430148 10.1016/0165-5728(92)90129-9

[29] Kotter MR, Setzu A, Sim FJ, Van Rooijen N, Franklin RJ (2001) Macrophage depletion impairs oligodendrocyte remyelination following lysolecithin-induced demyelination. Glia 35: 204–212. 11494411 10.1002/glia.1085

[30] Kotter MR, Zhao C, van Rooijen N, Franklin RJ (2005) Macrophage-depletion induced impairment of experimental CNS remyelination is associated with a reduced oligodendrocyte progenitor cell response and altered growth factor expression. Neurobiology of Disease 18: 166–175. 15649707 10.1016/j.nbd.2004.09.019

[31] Scherer SS, Salzer JL (2001) Axon–Schwann cell interactions during peripheral nerve degeneration and regeneration. In: Jessen KR, Richardson WD, editors. Glial Cell Development. Oxford: Oxford University Press.

[32] Koda M, Murakami M, Ino H, Yoshinaga K, Ikeda O, et al (2002) Brain-derived neurotrophic factor suppresses delayed apoptosis of oligodendrocytes after spinal cord injury in rats. Journal of Neurotrauma 19: 777–785. 12165137 10.1089/08977150260139147

[33] Huang EJ, Reichardt LF (2001) Neurotrophins: Roles in neuronal development and function. Annual Reviews Neuroscience 24: 677–736. 10.1146/annurev.neuro.24.1.677 PMC2758233 11520916

[34] Chan JR (2001) Inaugural Article: Neurotrophins are key mediators of the myelination program in the peripheral nervous system. Proceedings of the National Academy of Sciences USA 98: 14661–14668. 10.1073/pnas.251543398 PMC64738 11717413

[35] Chan JR, Jolicoeur C, Yamauchi J, Elliott J, Fawcett JP, et al (2006) The polarity protein Par-3 directly interacts with p75NTR to regulate myelination. Science 314: 832–836. 17082460 10.1126/science.1134069

[36] Cosgaya JM (2002) The neurotrophin receptor p75NTR as a positive modulator of myelination. Science 298: 1245–1248. 12424382 10.1126/science.1076595

[37] Mannion RJ, Costigan M, Decosterd I, Amaya F, Ma Q-P, et al (1999) Neurotrophins: Peripherally and centrally acting modulators of tactile stimulus-induced infalmmatory pain hypersensitivity. Proceedings of the National Academy of Sciences USA 96: 9385–9390. 10.1073/pnas.96.16.9385 PMC17792 10430952

[38] Tonra JR, Curtis R, Wong V, Cliffer KD, Park JS, et al (1998) Axotomy upregulates the anterograde transport and expression of brain-derived neurotrophic factor by sensory neurons. The Journal of Neuroscience 18: 4374–4383. 9592114 10.1523/JNEUROSCI.18-11-04374.1998 PMC6792814

[39] Zhou X-F, Chie ET, Deng Y-S, Zhong J-H, Xue Q, et al (1999) Injured primary sensory neurons switch phenotype for brain-derived neurotrophic factor in the rat. Neuroscience 92: 841–853. 10426526 10.1016/s0306-4522(99)00027-5

[40] Zhou X-F, Rush RA (1996) Endogenous brain-derived neurotrophic factor is anterogradely transported in primary sensory neurons. Neuroscience 74: 945–951. 8895863 10.1016/0306-4522(96)00237-0

[41] Ng BK, Chen L, Mandemakers W, Cosgaya JM, Chan JR (2007) Anterograde transport and secretion of brain-derived neurotrophic factor along sensory axons promote Schwann cell myelination. The Journal of Neuroscience 27: 7597–7603. 17626221 10.1523/JNEUROSCI.0563-07.2007 PMC6672611

[42] Meyer M, Matsuoka I, Wetmore C, Olson L, Thoenen H (1992) Enhanced synthesis of brain-derived neurotrophic factor in the lesioned peripheral nerve: Different mechanisms are responsible for the regulation of BDNF and NGF mRNA. The Journal of Cell Biology 119: 45–54. 1527172 10.1083/jcb.119.1.45 PMC2289627

[43] Zhang J-Y, Luo X-G, Xian CJ, Liu Z-H, Zhou X-F (2000) Endogenous BDNF is required for myelination and regeneration of injured sciatic nerve in rodents. European Journal of Neuroscience 12: 4171–4180. 11122329

[44] Barouch R, Appel E, Kazimirsky G, Brodie C (2001) Macrophages express neurotrophins and neurotrophin receptors: Regulation of nitric oxide production by NT-3. Journal of Neuroimmunology 112: 72–77. 11108935 10.1016/s0165-5728(00)00408-2

[45] Dougherty KD, Dreyfus CF, Black IB (2000) Brain-derived neurotrophic factor in astrocytes, oligodendrocytes, and microglia/macrophages after spinal cord injury. Neurobiology of Disease 7: 574–585. 11114257 10.1006/nbdi.2000.0318

[46] Griffin JW, Stocks EA, Fahnestock K, Van Praagh A, Trapp BD (1990) Schwann cell proliferation following lysolecithin-induced demyelination. Journal of Neurocytology 19: 367–384. 2391539 10.1007/BF01188405

[47] Rubinstein CT, Shrager P (1990) Remyelination of nerve fibres in the transected frog sciatic nerve. Brain Research 524: 303–312. 2292011 10.1016/0006-8993(90)90705-g

[48] McTigue DM, Horner PJ, Stokes BT, Gage FH (1998) Neurotrophin-3 and brain-derived neurotrophic factor induce oligodendrocyte proliferation and myelination of regenerating axons in the contused adult rat spinal cord. The Journal of Neuroscience 18: 5354–5365. 9651218 10.1523/JNEUROSCI.18-14-05354.1998 PMC6793495

[49] Wake H, Lee PR, Fields RD (2011) Control of local protein synthesis and initial events in myelination by action potentials. Science 333: 1647–1651. 21817014 10.1126/science.1206998 PMC3482340

[50] Al-Majed AA, Neumann CM, Brushart TM, Gordon T (2000) Brief electrical stimulation promotes the speed and accuracy of motor axonal regeneration. The Journal of Neuroscience 20: 2602–2608. 10729340 10.1523/JNEUROSCI.20-07-02602.2000 PMC6772244

[51] Back SA, Tuohy TM, Chen H, Wallingford N, Craig A, et al (2005) Hyaluronan accumulates in demyelinated lesions and inhibits oligodendrocyte progenitor maturation. Nature Medicine 11: 966–972. 10.1038/nm1279 16086023

[52] Brushart TM, Jari R, Verge V, Rohde C, Gordon T (2005) Electrical stimulation restores the specificity of sensory axon regeneration. Experimental Neurology 194: 221–229. 15899259 10.1016/j.expneurol.2005.02.007

[53] Geremia NM, Gordon T, Brushart TM, Al-Majed AA, Verge VMK (2007) Electrical stimulation promotes sensory neuron regeneration and growth-associated gene expression. Experimental Neurology 205: 347–359. 17428474 10.1016/j.expneurol.2007.01.040

[54] Singh B, Xu Q-G, Franz CK, Zhang R, Dalton C, et al (2012) Accelerated axon outgrowth, guidance, and target reinnervation across nerve transection gaps following a brief electrical stimulation paradigm. Journal of Neurosurgery 116: 498–512. 22149377 10.3171/2011.10.JNS11612

[55] Al-Majed AA, Brushart TM, Gordon T (2000) Electrical stimulation accelerates and increases expression of BDNF and trkB mRNA in regenerating rat femoral motoneurons. European Journal of Neuroscience 12: 4381–4390. 11122348

[56] English AW, Schwartz G, Meador W, Sabatier MJ, Mulligan A (2006) Electrical stimulation promotes peripheral axon regeneration by enhanced neuronal neurotrophin signaling. Developmental Neurobiology 67: 158–172. 10.1002/dneu.20339 PMC4730384 17443780

[57] Geremia NM, Pettersson LME, Hasmatali JC, Hryciw T, Danielsen N, et al (2010) Endogenous BDNF regulates induction of intrinsic neuronal growth programs in injured sensory neurons. Experimental Neurology 223: 128–142. 19646438 10.1016/j.expneurol.2009.07.022

[58] Hall SM (1972) The effect of injections of lysophosphatidyl choline into white matter of the adult mouse spinal cord. Journal of Cell Science 10: 535–546. 5018033 10.1242/jcs.10.2.535

[59] Hall SM, Gregson NA (1971) The in vivo and ultrastructural effects of injection of lysophosphatidyl choline into myelinated peripheral nerve fibres of the adult mouse. Journal of Cell Science 9: 769–789. 5148016 10.1242/jcs.9.3.769

[60] Allt G, Ghabriel MN, Sikri K (1988) Lysophosphatidyl choline-induced demyelination a freeze-fracture study. Acta Neuropathologica 75: 456–464. 3376751 10.1007/BF00687132

[61] Fitzgerald M (1987) Spontaneous and evoked activity of fetal primary afferents in vivo. Nature 326: 603–605. 3561499 10.1038/326603a0

[62] Schafer DP, Custer AW, Shrager P, Rasband MN (2006) Early events in node of Ranvier formation during myelination and remyelination in the PNS. Neuron Glia Biology 2: 69–79. 16652168 10.1017/S1740925X06000093 PMC1424668

[63] Kuhlmann T, Bitsch A, Stadelmann C, Siebert H, Bruck W (2001) Macrophages are eliminated from the injured peripheral nerve via local apoptosis and circulation to regional lymph nodes and the spleen. The Journal of Neuroscience 21: 3401–3408. 11331370 10.1523/JNEUROSCI.21-10-03401.2001 PMC6762479

[64] Martini R, Fischer S, López-Vales R, David S (2008) Interactions between Schwann cells and macrophages in injury and inherited demyelinating disease. Glia 56: 1566–1577. 18803324 10.1002/glia.20766

[65] Dieni S, Matsumoto T, Dekkers M, Rauskolb S, Ionescu MS, et al (2012) BDNF and its pro-peptide are stored in presynaptic dense core vesicles in brain neurons. The Journal of Cell Biology 196: 775–788. 22412021 10.1083/jcb.201201038 PMC3308691

[66] Stevens B, Tanner SL, Fields RD (1998) Control of myelination by specific patterns of neural impulses. The Journal of Neuroscience 18: 9303–9311. 9801369 10.1523/JNEUROSCI.18-22-09303.1998 PMC6792896

[67] Ishibashi T, Dakin KA, Stevens B, Lee PR, Kozlov SV, et al (2006) Astrocytes Promote Myelination in Response to Electrical Impulses. Neuron 49: 823–832. 16543131 10.1016/j.neuron.2006.02.006 PMC1474838

[68] Gibson EM, Purger D, Mount CW, Goldstein AK, Lin GL, et al (2014) Neuronal activity promotes oligodendrogenesis and adaptive myelination in the mammalian brain. Science 344: 1252304. 24727982 10.1126/science.1252304 PMC4096908

[69] Wan L, Zhang S, Xia R, Ding W (2010) Short-term low-frequency electrical stimulation enhanced remyelination of injured peripheral nerves by inducing the promyelination effect of brain-derived neurotrophic factor on Schwann cell polarization. Journal of Neuroscience Research 88: 2578–2587. 20648648 10.1002/jnr.22426

[70] Pavelko KD, van Engelen BGM, Rodriguez M (1998) Acceleration in the rate of CNS remyelination in lysolecithin-induced demyelination. The Journal of Neuroscience 18: 2498–2505. 9502810 10.1523/JNEUROSCI.18-07-02498.1998 PMC6793082

[71] Scholz J, Klein MC, Behrens TE, Johansen-Berg H (2009) Training induces changes in white-matter architecture. Nature Neuroscience 12: 1370–1371. 19820707 10.1038/nn.2412 PMC2770457

[72] Sampaio-Baptista C, Khrapitchev AA, Foxley S, Schlagheck T, Scholz J, et al (2013) Motor skill learning induces changes in white matter microstructure and myelination. The Journal of Neuroscience 33: 19499–19503. 24336716 10.1523/JNEUROSCI.3048-13.2013 PMC3858622

[73] Verderio C, Bianco F, Blanchard MP, Bergami M, Canossa M, et al (2007) Cross talk between vestibular neurons and Schwann cells mediates BDNF release and neuronal regeneration. Brain Cell Biology 35: 187–201. 10.1007/s11068-007-9011-6 17957483

[74] Bouhy D, Malgrange B, Multon S, Poirrier AL, Scholtes F, et al (2006) Delayed GM-CSF treatment stimulates axonal regeneration and functional recovery in paraplegic rats via an increased BDNF expression by endogenous macrophages. The FASEB Journal 20: 1239–1241. 16636109 10.1096/fj.05-4382fje

[75] Wong I, Liao H, Bai X, Zaknic A, Zhong J, et al (2010) ProBDNF inhibits infiltration of ED1+ macrophages after spinal cord injury. Brain, Behavior, and Immunity 24: 585–597. 10.1016/j.bbi.2010.01.001 20083190

[76] Michael GJ, Averill S, Nitkunan A, Rattray M, Bennett DLH, et al (1997) Nerve growth factor treatment increases brain-derived neurotrophic factor selectively in TrkA-expressing dorsal root ganglion cells and in their central terminations within the spinal cord. The Journal of Neuroscience 17: 8476–8490. 9334420 10.1523/JNEUROSCI.17-21-08476.1997 PMC6573719

[77] Bernardini N, Neuhuber W, Reeh PW, Sauer SK (2004) Morphological evidence for functional capsaicin receptor expression and calcitonin gene-related peptide exocytosis in isolated peripheral nerve axons of the mouse. Neuroscience 126: 585–590. 15183508 10.1016/j.neuroscience.2004.03.017

[78] Sauer SK, Bove GM, Averbeck B, Reeh PW (1999) Rat peripheral nerve components release calcitonin gene-related peptide and prostaglandin E2 in response to noxious stimuli: Evidence that nervi nervorum are nociceptors. Neuroscience 92: 319–325. 10392853 10.1016/s0306-4522(98)00731-3

[79] Tolwani RJ, Cosgaya JM, Varma S, Jacob R, Kuo LE, et al (2004) BDNF overexpression produces a long-term increase in myelin formation in the peripheral nervous system. Journal of Neuroscience Research 77: 662–669. 15352212 10.1002/jnr.20181

[80] English AW, Liu K, Nicolini JM, Mulligan AM, Ye K (2013) Small-molecule trkB agonists promote axon regeneration in cut peripheral nerves. Proceedings of the National Academy of Sciences USA 110: 16217–16222. 10.1073/pnas.1303646110 PMC3791704 24043773

[81] Einheber S, Zanazzi G, Ching W, Scherer SS, Milner TA, et al (1997) The axonal membrane protein Caspr, a homologue of neurexin IV, is a component of the septate-like paranodal junctions that assemble during myelination. The Journal of Cell Biology 139: 1495–1506. 9396755 10.1083/jcb.139.6.1495 PMC2132621

[82] Cui QL, Fragoso G, Miron VE, Darlington PJ, Mushynski WE, et al (2010) Response of human oligodendrocyte progenitors to growth factors and axon signals. Journal of Neuropathology and Experimental Neurology 69: 930–944. 20720504 10.1097/NEN.0b013e3181ef3be4

[83] Demerens C, Stankoff B, Logak M, Anglade P, Allinquant B, et al (1996) Induction of myelination in the central nervous system by electrical activity. Proceedings of the National Academy of Sciences USA 93: 9887–9892. 10.1073/pnas.93.18.9887 PMC38524 8790426

[84] Colello RJ, Devey LR, Imperato E, Pott U (1995) The chronology of oligodendrocyte differentiation in the rat optic nerve: Evidence for a signaling step initiating myelination in the CNS. The Journal of Neuroscience 15: 7665–7672. 7472517 10.1523/JNEUROSCI.15-11-07665.1995 PMC6578053

[85] Dyck PJ, Karnes JL, Lambert EH (1989) Longitudinal study of neuropathic deficits and nerve conduction abnormalities in hereditary motor and sensory neuropathy type 1. Neurology 39: 1302–1308. 2797453 10.1212/wnl.39.10.1302

[86] Yagihashi S, Kamijo M, Watanabe K (1990) Reduced myelinated fiber size correlates with loss of axonal neurofilaments in peripheral nerve of chronically streptozotocin diabetic rats. The American Journal of Pathology 136: 1365–1373. 2141449 PMC1877565

[87] Tokuoka H, Saito T, Yorifuji H, Wei F-Y, Kishimoto T, et al (2000) Brain-derived neurotrophic factor-induced phosphorylaton of neurofilament-H subunit in primary cultures of embryo rat cortical neurons. Journal of Cell Science 113: 1059–1068. 10683153 10.1242/jcs.113.6.1059

[88] Weinberg HJ, Spencer PS (1976) Studies on the control of myelinogenesis II: Evidence for neuronal regulation of myelin production. Brain Research 113: 363–378. 953741 10.1016/0006-8993(76)90947-1

[89] Chan JR, Watkins TA, Cosgaya JM, Zhang C, Chen L, et al (2004) NGF controls axonal receptivity to myelination by Schwann cells or oligodendrocytes. Neuron 43: 183–191. 15260955 10.1016/j.neuron.2004.06.024 PMC2758239

[90] Camara J, Wang Z, Nunes-Fonseca C, Friedman HC, Grove M, et al (2009) Integrin-mediated axoglial interactions initiate myelination in the central nervous system. The Journal of Cell Biology 185: 699–712. 19451276 10.1083/jcb.200807010 PMC2711572

[91] Kiefer R, Kieseier BC, Stoll G, Hartung H-P (2001) The role of macrophages in immune-mediated damage to the peripheral nervous system. Progress in Neurobiology 64: 109–127. 11240209 10.1016/s0301-0082(00)00060-5

[92] Fry EJ, Ho C, David S (2007) A role for Nogo receptor in macrophage clearance from injured peripheral nerve. Neuron 53: 649–662. 17329206 10.1016/j.neuron.2007.02.009

[93] Wang J, Zhang P, Wang Y, Kou Y, Zhang H, et al (2010) The observation of phenotypic changes of Schwann cells after rat sciatic nerve injury. Artificial Cells, Blood Substitutes and Biotechnology 38: 24–28. 10.3109/10731190903495736 20047518

[94] Cheng C, Zochodne DW (2002) In vivo proliferation, migration and phenotypic changes of schwann cells in the presence of myelinated fibers. Neuroscience 115: 321–329. 12401344 10.1016/s0306-4522(02)00291-9

[95] Clerico M, Rivoiro C, Contessa G, Viglietti D, Durelli L (2008) The therapy of multiple sclerosis with immune-modulating or immunosuppressive drug. Clinical Neurology and Neurosurgery 110: 878–885. 18164542 10.1016/j.clineuro.2007.10.020

[96] Nakahara J, Aiso S, Suzuki N (2009) Factors that retard remyelination in multiple sclerosis with a focus on TIP30: a novel therapeutic target. Expert Opinion on Therapeutic Targets 13: 1375–1386. 19839715 10.1517/14728220903307491

