# Supplementary figures and images for: Delayed Nerve Stimulation Promotes Axon-Protective Neurofilament Phosphorylation, Accelerates Immune Cell Clearance and Enhances Remyelination In Vivo in Focally Demyelinated Nerves
Source: PLoS One. 2014 Oct 13;9(10):e110174. doi: 10.1371/journal.pone.0110174 (PMC4195712; doi:10.1371/journal.pone.0110174)

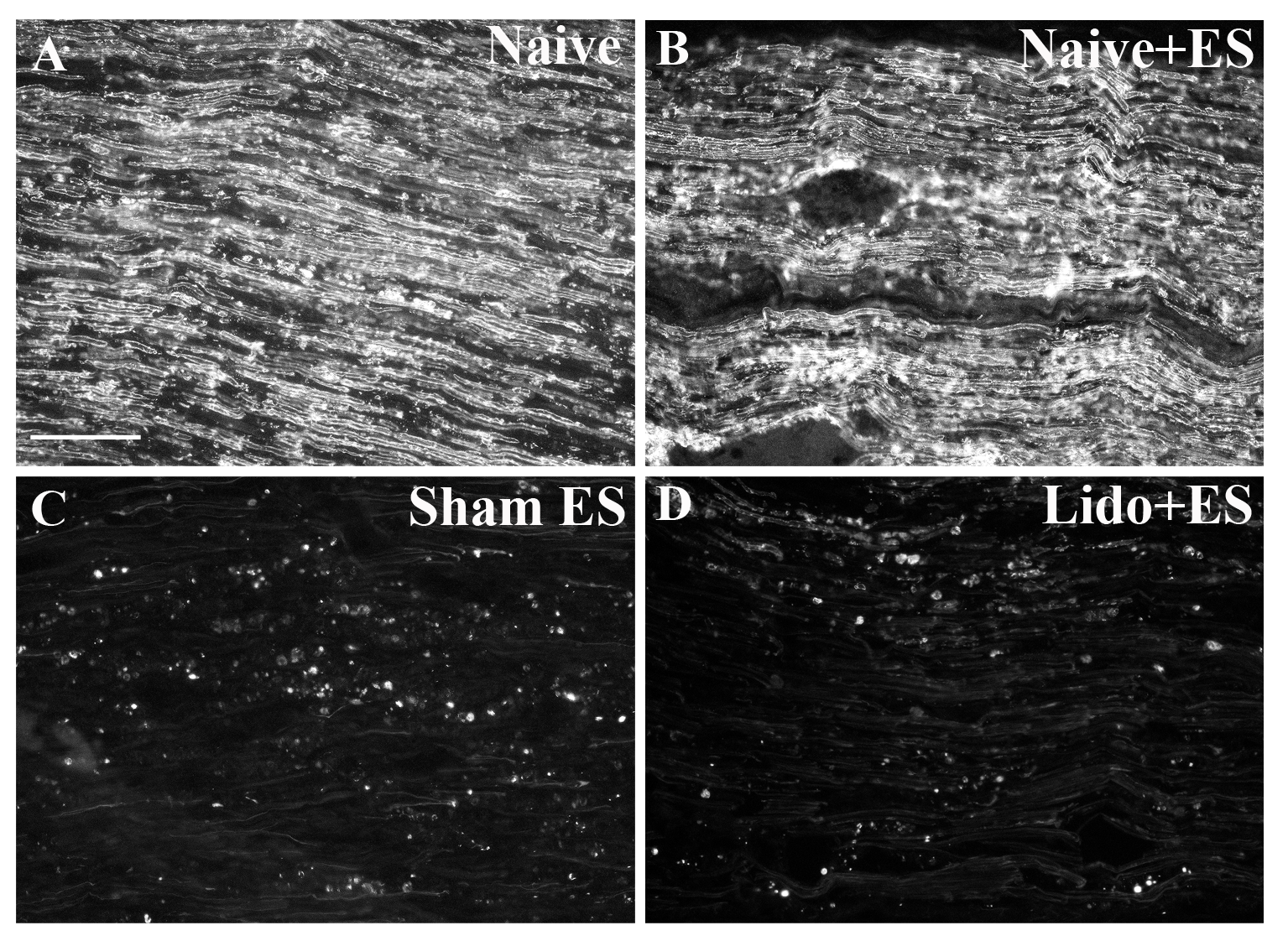

Supplement: Figure S1 — Increased neuronal activity effects increases in myelin basic protein (MBP) observed following electrical stimulation of focally demyelinated nerve. Representative immunofluorescence photomicrographs of tibial branch of sciatic nerve sections immunostained for MBP. Naïve (uninjured) nerves displayed intense MBP immunoreactivity (A). Electrical stimulation (ES) of uninjured nerves appeared to have no affect on the integrity of the myelin sheath (B). Sham ES and blockage of action potential conduction through local application of lidocaine did not result in the increased remyelination following unilateral focal LPC demyelination normally observed upon application of ES (C, D). This indicates that the increased MBP immunoreactivity observed in electrically stimulated nerves is related to the axonal activity induced by the ES procedure. Scale bar = 100 µm. (TIF) [file pone.0110174.s001.tif]

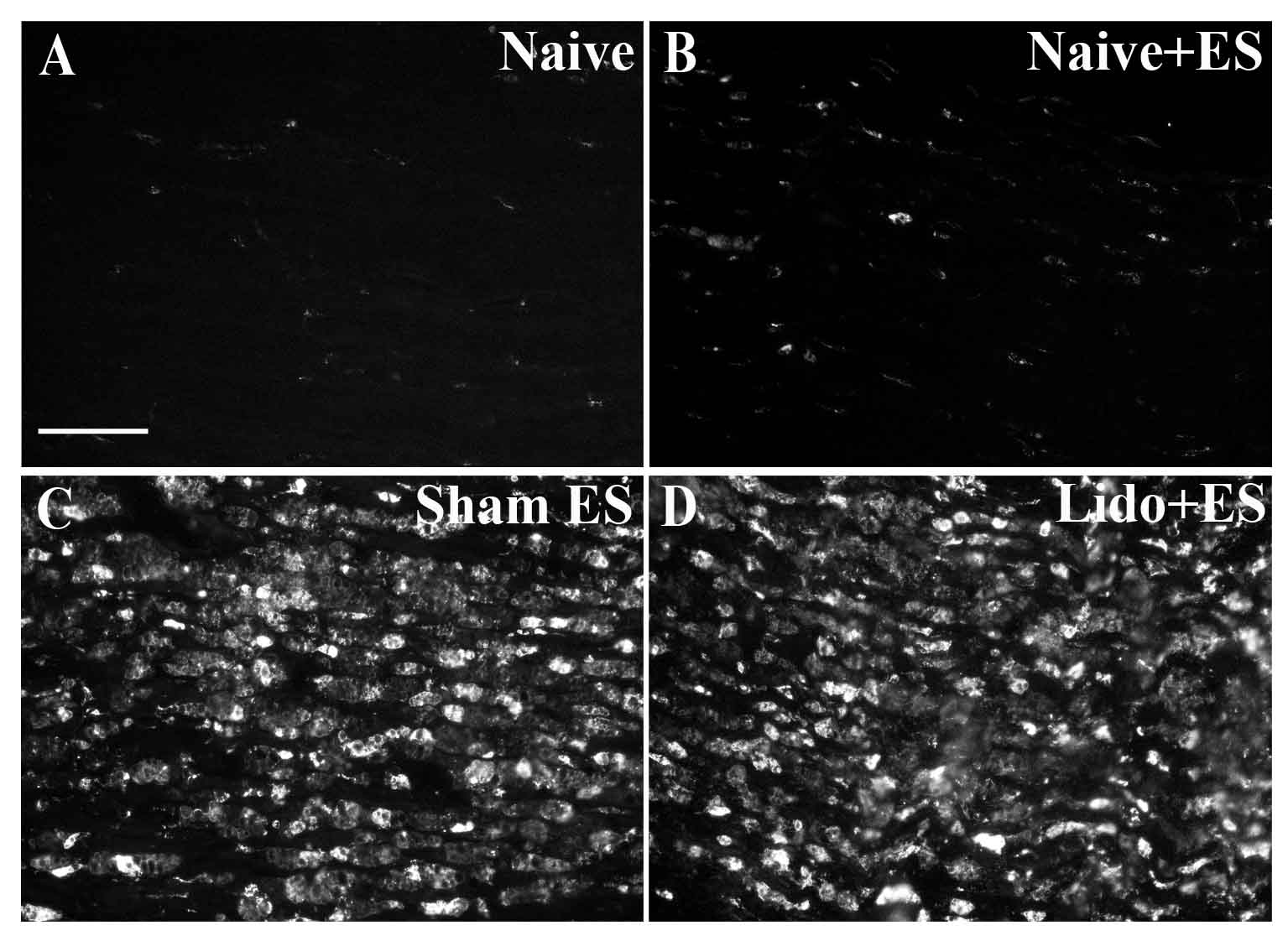

Supplement: Figure S2 — Increased neuronal activity is required to effect reductions in activated macrophage (ED1) immunoreactivity in focally demyelinated regions. Representative immunofluorescence photomicrographs of tibial nerve sections immunostained for ED1. Naïve (uninjured) nerves displayed minimal ED1 immunoreactivity (A). Electrical stimulation (ES) alone did not trigger an inflammatory response nor an infiltration of activated macrophages into the stimulation site (B). Sham stimulation and blockade of action potential conduction through local application of lidocaine did not result in an increase in macrophage clearance from the lesion site (C, D). This indicates that the enhanced clearance of macrophages from the demyelinated lesions of animals receiving electrical stimulation (ES) is related to the activity induced by the ES procedure. Scale bar = 100 µm. (TIF) [file pone.0110174.s002.tif]

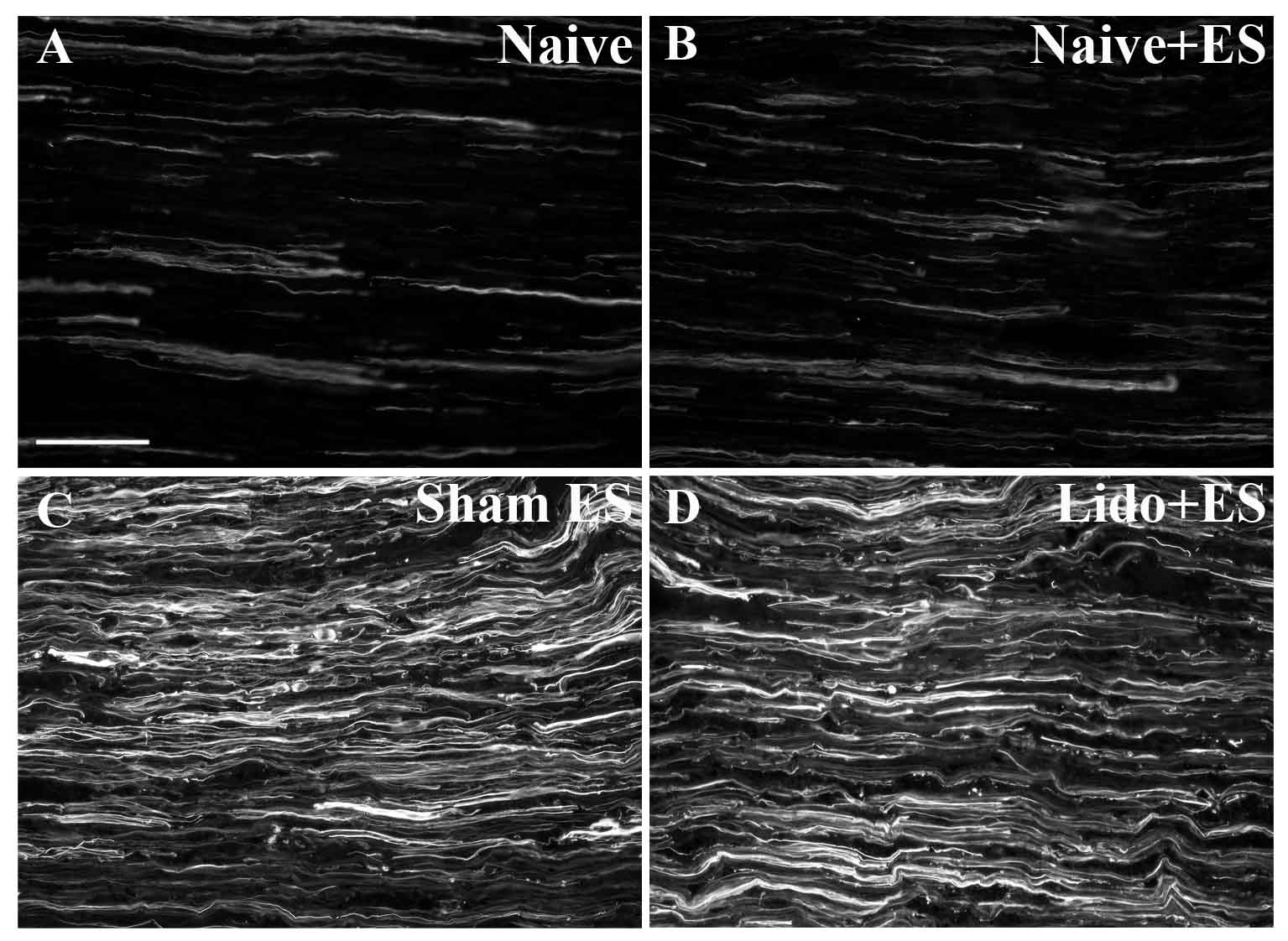

Supplement: Figure S3 — Increased neuronal activity is required to effect a reduction in Schwann cell reactivity in zones of focal demyelination. Representative immunofluorescence photomicrographs (20x magnification) of sciatic nerve sections immunostained for glial fibrillary acidic protein (GFAP). Naïve (uninjured) nerves display some GFAP immunoreactivity, reflecting the population of non-myelinating Schwann cells present within peripheral nerves (A). Electrical stimulation alone did not induce reactive gliosis (B). Sham stimulation and blockage of action potential conduction through local application of lidocaine did not result in a decrease in reactive gliosis within the lesion site (C, D). This indicates that the reduction in Schwann cell reactivity observed within the demyelinated lesions of animals receiving electrical stimulation is related to the activity induced by the electrical stimulation procedure. Scale bar = 100 µm. (TIF) [file pone.0110174.s003.tif]

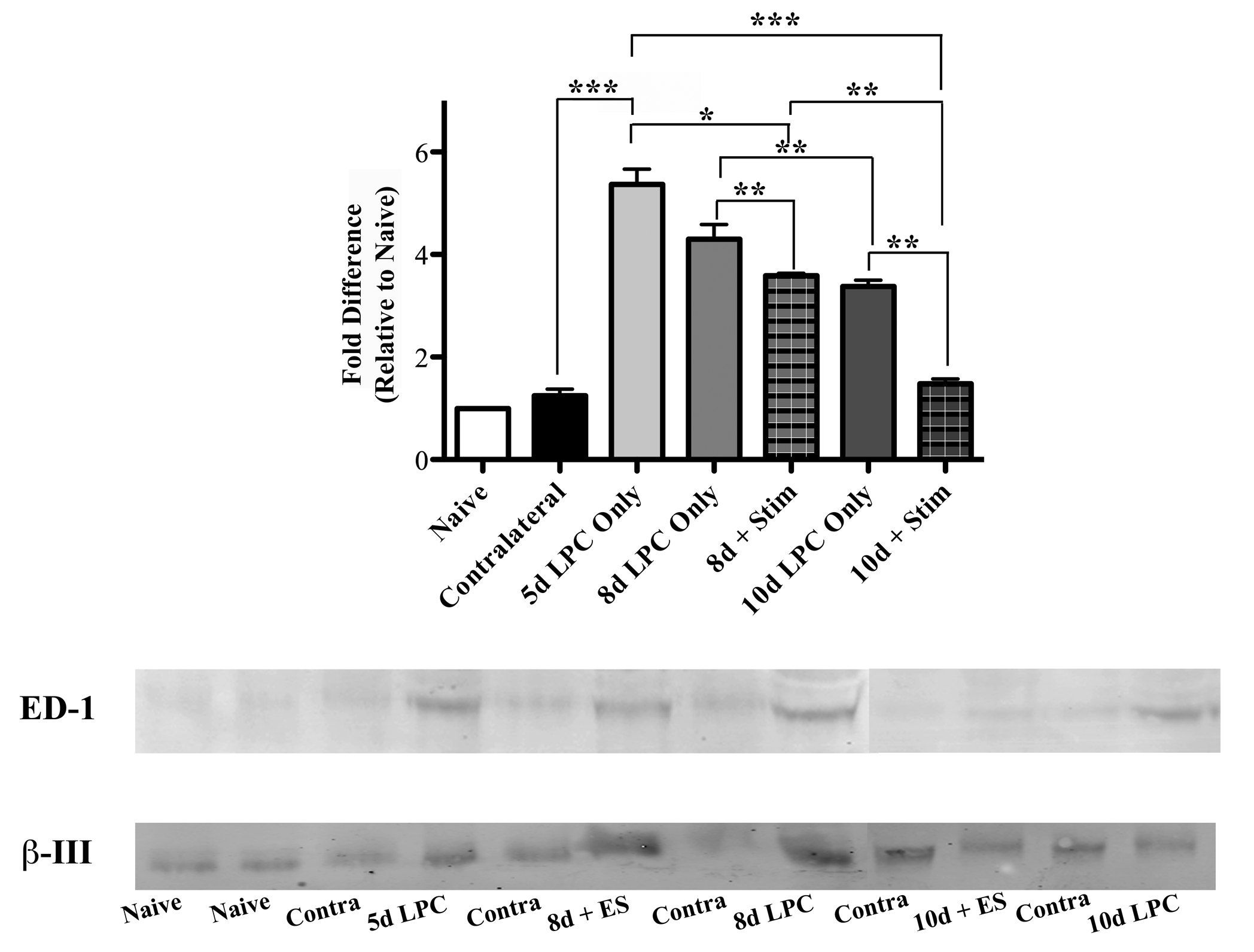

Supplement: Figure S4 — 1hr electrical stimulation (ES) 5 days post-lysophosphatidyl choline (LPC) results in decreased ED-1 content beginning 8d post-LPC. Representative Western blots of sciatic nerve extract probed for ED-1. Immunoblots were run in duplicate from pooled nerve samples from 3 animals/experimental condition. Densitometry readings were normalized to the loading control β-III tubulin within each lane and compared to the mean densitometry reading of the two lanes of naïve sciatic nerve protein extract run alongside the demyelinated nerve extracts in each gel. There was a marked increase in detectable ED-1 observed 5d post-lysophosphatidyl choline (LPC) injection into the tibial branch of the sciatic nerve, as compared to that observed in protein extract from both naïve and contralateral (uninjured) nerves. As early as three days post-ES (8d post-LPC), there was a decrease in the amount of detectable ED-1 in the stimulated nerves. This drop preceded the visual differences observed immunohistochemically (Figure 4J, K). Levels of detectable ED-1 showed further decline 5d post-ES (10d post-LPC), where they reached levels not significantly different than that of the naïve or contralateral (uninjured) controls. Asterisks indicate significant differences between experimental groups; *P<0.05, **P<0.01, ***P<0.001, Student's t-test. (TIF) [file pone.0110174.s004.tif]

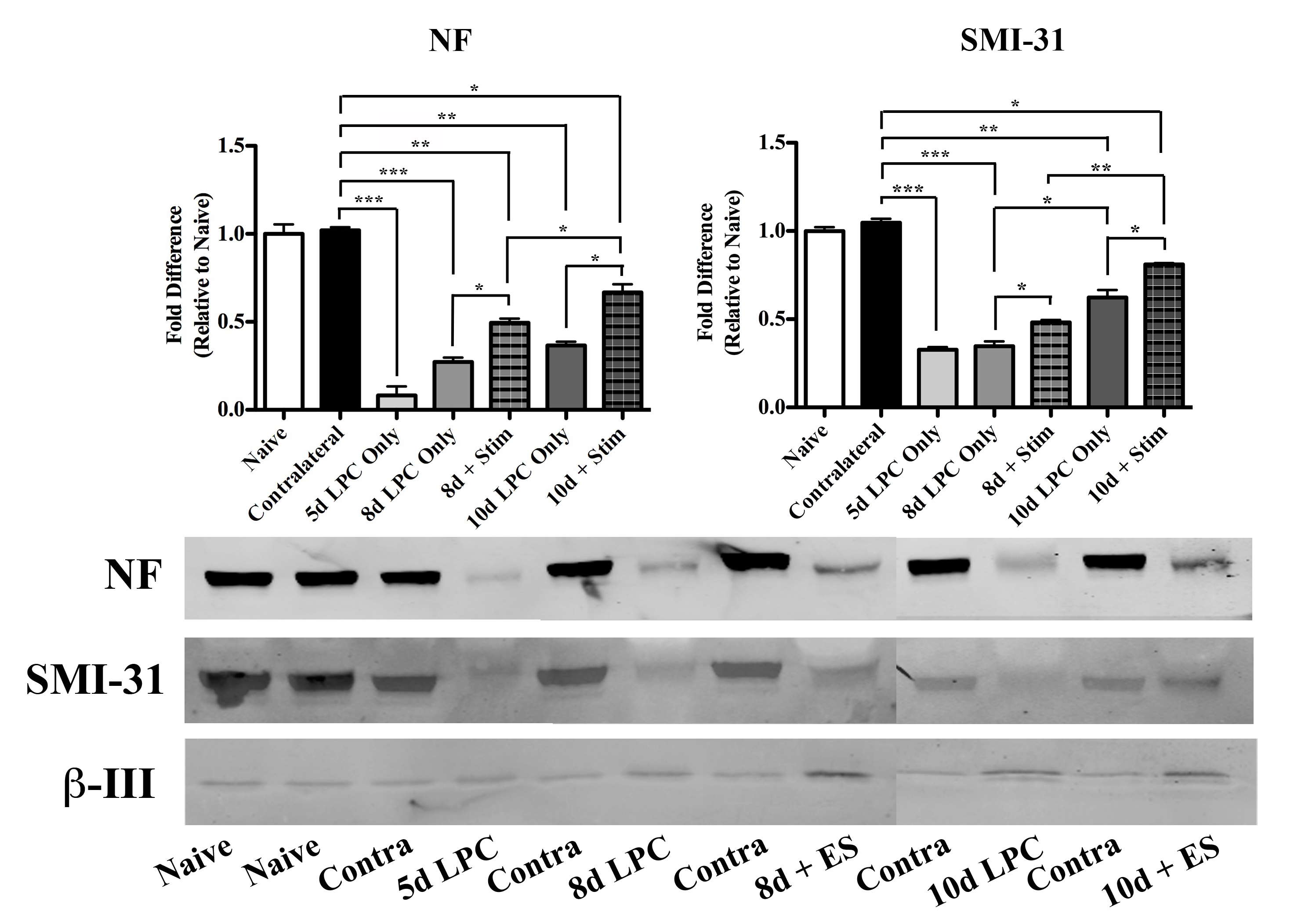

Supplement: Figure S5 — 1hr brief electrical stimulation (ES) results in increased expression of total and phosphorylated neurofilament proteins. Representative Western blot of tibial nerve demyelination zone protein extract and probed for total (NF) and phosphorylated neurofilaments (SMI-31). Immunoblots were run in duplicate from pooled nerve samples from 3 animals/experimental condition. Densitometry readings were normalized to the loading control β-III tubulin within each lane and compared to the mean densitometry reading of the two lanes of naïve sciatic nerve protein extract run alongside the demyelinated nerve extracts in each gel. Naïve and contralateral control nerves displayed intense NF and SMI-31 immunoreactivity. There was a marked decrease in both NF and SMI-31 band intensity observed 5d post-lysophosphatidyl choline (LPC)/FG injection into the tibial branch of the sciatic nerve. ES resulted in an increase in the amount of detectable NF and SMI-31 proteins 3 and 5 days post-ES (8 and 10 days post-LPC), consistent with the immunohistochemical observations and quantification (see Figure 5). In focally demyelinated nerves that did not undergo ES the levels of NF and SMI-31 remained low. Asterisks indicate significant differences between experimental groups; *P<0.05, **P<0.01, ***P<0.001, Student's t-test. (TIF) [file pone.0110174.s005.tif]
